# Application of In Vitro Models for Studying the Mechanisms Underlying the Obesogenic Action of Endocrine-Disrupting Chemicals (EDCs) as Food Contaminants—A Review

**DOI:** 10.3390/ijms24021083

**Published:** 2023-01-05

**Authors:** Monika Kowalczyk, Jakub P. Piwowarski, Artur Wardaszka, Paulina Średnicka, Michał Wójcicki, Edyta Juszczuk-Kubiak

**Affiliations:** 1 Laboratory of Biotechnology and Molecular Engineering, Department of Microbiology, Prof. Wacław Dąbrowski Institute of Agricultural and Food Biotechnology—State Research Institute, 02-532 Warsaw, Poland; 2 Microbiota Lab, Department of Pharmacognosy and Molecular Basis of Phytotherapy, Medical University of Warsaw, 02-097 Warsaw, Poland

**Keywords:** food contaminants, endocrine-disrupting chemicals (EDCs), obesogens, in vitro models, adipose tissue, liver, endocrine pancreas, obesity, metabolism disorder

## Abstract

Obesogenic endocrine-disrupting chemicals (EDCs) belong to the group of environmental contaminants, which can adversely affect human health. A growing body of evidence supports that chronic exposure to EDCs can contribute to a rapid increase in obesity among adults and children, especially in wealthy industrialized countries with a high production of widely used industrial chemicals such as plasticizers (bisphenols and phthalates), parabens, flame retardants, and pesticides. The main source of human exposure to obesogenic EDCs is through diet, particularly with the consumption of contaminated food such as meat, fish, fruit, vegetables, milk, and dairy products. EDCs can promote obesity by stimulating adipo- and lipogenesis of target cells such as adipocytes and hepatocytes, disrupting glucose metabolism and insulin secretion, and impacting hormonal appetite/satiety regulation. In vitro models still play an essential role in investigating potential environmental obesogens. The review aimed to provide information on currently available two-dimensional (2D) in vitro animal and human cell models applied for studying the mechanisms of obesogenic action of various industrial chemicals such as food contaminants. The advantages and limitations of in vitro models representing the crucial endocrine tissue (adipose tissue) and organs (liver and pancreas) involved in the etiology of obesity and metabolic diseases, which are applied to evaluate the effects of obesogenic EDCs and their disruption activity, were thoroughly and critically discussed.

## 1. Introduction

According to the World Health Organization (WHO), obesity is one of the top ten threats to human health [1]. The research of the Statistical Office of the European Union (Eurostat) shows that 52.7% of the adult population of the European Union (EU) was overweight in 2019, of which approximately 17% were obese [2]. Moreover, in the EU, every eighth child aged 7–8 is obese [3]. Nowadays, there are far more people in the world overweight or classified as obese than malnourished and obesity has become a serious health problem in both developed and developing countries [1]. Obesity is associated with comorbidities such as the increased risk of cardiovascular disease, insulin resistance, Type 2 diabetes mellitus (T2DM), hypertension, as well as non-alcoholic fatty liver disease (NAFLD), and hormone-sensitive cancers [4]. Obesity is characterized by an imbalance between energy intake and total energy expenditure resulting in increased lipid accumulation in adipocytes and excess fat storage in the body [5,6]. However, recent studies have demonstrated that consumption of a calorie-dense diet coupled with physical inactivity as well as genetics cannot explain rising obesity among adults and children [7,8]. Over the past decade, there is considerable evidence that a substantial increase in environmental chemical production may contribute to the rapid increase in human obesity and metabolic syndrome [7,8]. Especially, environmentally existing xenobiotic chemicals, such as endocrine-disrupting chemicals (EDCs) are the main candidates [4,9,10,11,12,13]. EDCs are a class of natural or synthetic exogenous chemical substances that may interfere with the function of the endocrine system by mimicking or blocking hormone biosynthesis, metabolism or action [4,7,9,10,13]. Their adverse effects on estrogen, androgen, and thyroid hormone signaling have been well documented [14,15,16,17]. Moreover, EDCs promote adverse effects during fetal life, infancy, puberty, and pregnancy via epigenetic mechanisms and some of these effects can be transmitted to the next generations [11,18,19,20]. Research shows that prenatal and perinatal exposure to EDCs may contribute to the greater storage of fat in the organism from the beginning of its life [13].

EDCs encompass a variety of chemical classes, including plasticizers, pesticides, industrial by-products, and pollutants [4,11,12]. The most common contaminants are bisphenols, phthalates, dioxins, pesticides, and polychlorinated biphenyls to which humans are exposed daily by eating, breathing polluted air, and drinking contaminated water [21]. However, new evidence has shown that some of them can stimulate lipid accumulation in target cells such as adipocytes and hepatocytes or disrupt sensitive metabolic processes, leading to obesity and metabolic syndrome [22]. These EDCs are referred to as “environmental obesogens” [9,18,23,24]. It has been reported that obesogens can promote obesity by increasing body weight [25], as well as causing hypertrophy and/or hyperplasia of adipocytes [26], stimulating the adipogenesis process [27], disturbing lipid and glucose metabolism [28], interfering with neuroendocrine regulation of satiety and appetite [7,28], inhibiting energy expenditure and/or brown adipose tissue thermogenesis [29], promoting inflammation [30], as well as changing in the taxonomic and metabolomic profiles of the gut microbiota [31]. The main source of human exposure to obesogenic EDCs is through diet, particularly the consumption of contaminated food such as meat, fish, fruit, vegetables, milk, and dairy products [21]. Such compounds can penetrate foods as a result of migration from food contact materials, including plastic containers for foodstuff and drinks and lining materials for food and beverage cans [32,33,34]. Exposure to these substances may also occur via inhalation and dermal absorption as they are widely used in personal care products (cosmetics, perfumes, lotions), detergents, medical devices, children’s toys, printing inks, the thermal paper used in cash register receipts as well as textiles [33,34,35]. Moreover, many chemicals that have been identified as obesogenic EDCs are pesticides, although some of them were withdrawn from general use many years ago but are still present in the environment [35,36]. EDCs’ concentration in food varies among countries and different foodstuffs as well as types of food packaging [37]. Moreover, socio-demographic, lifestyle and dietary habits can play important role in EDC exposure [38]. To date, there are more than 1000 chemicals reported to have endocrine effects [18,20,39] among which about 10 different classes of obesogenic chemicals including pesticides, in particular, organotins [40], industrial chemicals such as bisphenol A (BPA) [41] and di(2-ethylhexyl) phthalate (DEHP) [42], as well as parabens (alkyl esters of p-hydroxybenzoic acid) [43], have been characterized [44]. Several epidemiological trials have shown that high levels of these contaminants have been detected globally in human blood plasma [45], urine [46,47], breast milk [48,49], and adipose tissue [50]. Moreover, a significantly higher level of EDC exposure was observed in children compared to adults [38,51]. In Poland, results of a cohort study including children from the prospective Polish Mother and Child Cohort (REPRO_PL) showed a higher level of urinary phthalate metabolites in children at an age of 7 years than in the same children at age 2 and their mothers during pregnancy [51]. Moreover, phthalate exposures were much higher than exposures reported in other European children populations [51].

The dramatically increasing production of highly processed foods and evidence of health risks linked with food chemical contamination has led to the development of different chemical analogous and alternatives [52,53,54,55,56,57,58]. Nonetheless, results of several studies have suggested that some of the biological activities of chemical analogous are of a similar or even higher magnitude in comparison to initial compounds [58,59,60,61] but mechanistic aspects underlying the obesogenic effects remain largely unrecognized [62]. Therefore, in vitro models still play an essential role in identifying environmental obesogens and understanding obesogenic mechanisms for further insight into the in vivo action of these chemicals linked with the risk of developing various chronic diseases including obesity and metabolic disorders. Moreover, in vitro models provide a rapid approach to investigating hazards of exposure to EDCs and their toxicity potential, while reducing or eliminating the need for animal testing [63].

Hence, this review aimed to provide information from peer-reviewed literature on currently available 2D in vitro animal and human cell models applied for studying the mechanisms of action of the various obesogenic EDCs as food contaminants. We focused mainly on in vitro models representing the main tissues and organs involved in the etiology of obesity and metabolic diseases, such as adipose tissue, liver, and endocrine pancreas. These in vitro models are currently used to evaluate the chemical toxicity of EDCs and their metabolic disruption activity. We believe that this comprehensive insight could help scientists choose the appropriate in vitro model to study the mechanisms of action of environmental obesogens associated with the risk of human obesity and its comorbidities.

## 2. Adipogenesis and Obesogenic Action of Endocrine-Disrupting Chemicals (EDCs)

Adipose tissue (AT) is a metabolic active tissue located in the body under the skin (subcutaneous adipose tissue (SAT)) as well as around the internal organs (visceral adipose tissue (VAT)) [64]. AT contains different types of cells, such as preadipocytes, adipocytes, immune cells, fibroblasts, and endothelial cells [65]. In mammals, there are two types of AT, white (white adipose tissue (WAT)) and brown (brown adipose tissue (BAT)), which differ in anatomical location, morphology, functions, biochemical features, and gene expression patterns [66]. WAT accounts for over 95% of adipose mass, while BAT represents about 1–2% [66]. WAT is responsible for energy storage in triglycerides, formed from the esterification of glycerol-3-phosphate and free fatty acids (FFAs) [64]. The most dominant characteristic of BAT is non-shivering thermogenesis, where energy derived from fatty acid (FA) oxidation generates heat by mitochondrial uncoupling to maintain body temperature [29,66]. The magnitude of adipose tissue mass is determined by the enlargement of existing adipocytes (hypertrophy) and by an increase in preadipocyte number (hyperplasia) [64,67,68]. During childhood and adolescence, the number of adipocytes is determined and remains constant in the adult body, regardless of whether the individual is obese or lean [65]. In adulthood, the mass of WAT increases through hypertrophy [65]. In overnutrition, there is an increased accumulation of fat in the adipocytes and cells undergo cellular hypertrophy. In contrast, lipolysis occurs in the adipocytes during calorie restriction [68].

Adipogenesis is a multi-step process leading to the conversion of mesenchymal stem cells (MSCs) and preadipocytes into mature adipocytes and consists of three stages: commitment of MSCs to the adipocyte lineage, mitotic clonal expansion (replication of DNA and duplication of cells intensively takes place), and terminal differentiation [66]. Peroxisome proliferator-activated receptor γ (PPARγ) and CCAAT/enhancer-binding proteins (C/EBP), C/EBPδ, and C/EBPβ, are key transcription factors during the early stages of differentiation [66]. PPARγ and C/EBPα cooperatively promote differentiation and the induction of adipocyte-specific genes including, inter alia, adipocyte protein 2 (AP2) and glucose transporter type 4 (GLUT4). At the terminal differentiation stage, the preadipocytes acquire the features of mature adipocytes, such as insulin sensitivity, lipid synthesis and transport, and secretion of adipocyte-specific proteins [68].

Recent evidence showed that chronic human exposure to obesogenic EDCs can be associated with inducing preadipocyte differentiation, increasing oxidative stress, and promoting a pro-inflammatory state leading to an increase in the risk of obesity and metabolic disorders [7,8,18,69]. Numerous obesogens such as tributyltin (TBT), bisphenol A (BPA) as well as mono-2-ethylhexyl phthalate (MEHP) activate adipogenesis by acting on nuclear receptors (NRs), in particular by activating retinoid X receptor (RXR)/PPARγ-dependent signalling [70,71,72,73]. Activation of RXR/PPARγ plays a crucial role in the regulation of the expression of genes involved in lipid droplet formation, glucose uptake and insulin responsiveness [74]. Furthermore, obesogenic EDCs can promote adipogenesis and lipid storage and fat deposition by interfering with steroid hormone receptors such as glucocorticoid receptors (GRs) and estrogen receptors (ERs) [75,76]. For example, BPA-binding GRs directly increase adipogenesis and lipid accumulation and indirectly via induction of the 11beta-hydroxysteroid dehydrogenase 1 (*HSD11B1*) mRNA expression involved in cortisone/cortisol conversion [76].

**Figure 1:**
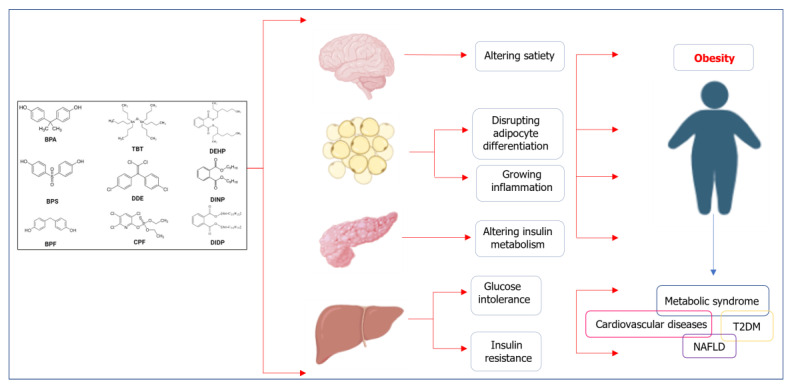
Summary of the effects of daily exposed obesogenic EDCs on crucial organs (liver and pancreas) and tissues (adipose and brain) and the relationship of these effects with human obesity and diabetes. T2DM, Type 2 diabetes mellitus; NAFLD, non-alcoholic fatty liver disease.

In vitro studies have shown that many obesogenic EDCs not only induce the differentiation of MSCs into adipocytes [55,77,78,79] but also alter the metabolism of mature adipocytes [8,62,80,81,82,83,84]. Exposure to EDCs can reduce the sensitivity of adipocytes to insulin, which causes an increase in blood glucose level and, consequently, may lead to insulin resistance in WAT of adulthood, potentially via a reduction in protein (serine/threonine) kinase B (PKB, also known as Akt) and GLUT4 levels [8,85]. Moreover, EDCs affect the expression of genes related to the de novo synthesis of free fatty acids, such as fatty acid synthase (*FASN*) or sterol regulatory element-binding protein 1c (*SREBP1C*), as well as the synthesis of triglycerides, such as diacylglycerol acyltransferase 1 and 2 (*DGAT1* and *DGAT2*), leading to a disturbance in the adipose lipid metabolism [86]. Recent evidence showed that the obesogenic action of some EDCs is associated with disruption of appetite/satiety signaling and food preferences [18]. As an endocrine organ, WAT plays an integral role in maintaining global energy homeostasis by secretion of adipokines (leptin, adiponectin), which regulate global insulin sensitivity, satiety and inflammation [66,87]. Several EDCs have been shown to alter leptin levels in animal models, including dichlorodiphenyldichloroethylene (DDE) and DEHP [80,84]. The in vitro effect of EDCs on other adipokines such as resistin correlated with insulin signaling has also been reported [80]. The potential mechanisms of the obesogenic action of daily exposed EDCs on the development of obesity and metabolic syndrome are presented in Figure 1.

## 3. In vitro Models Used in the Study of the Obesogenic Effects of EDCs

**Figure 2:**
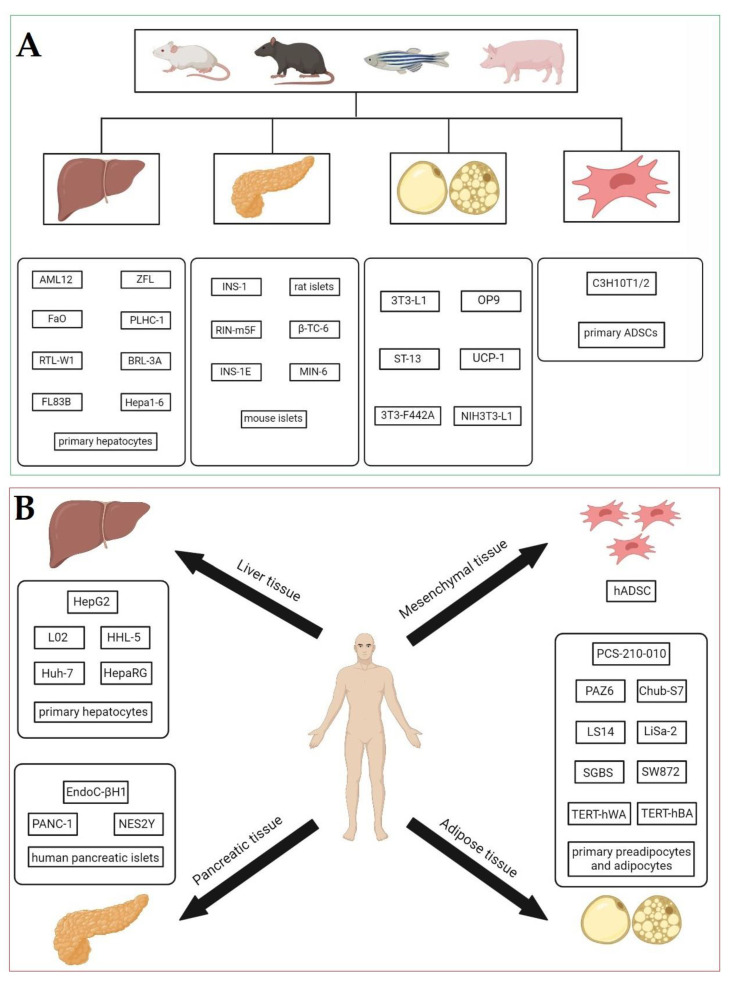
In vitro models applied in the study of the obesogenic action of endocrine-disrupting chemicals (EDCs): (**A**) animals, and (**B**) humans.

Data from epidemiological trials are essential for evaluating of potential adverse effects of EDC exposure but usually provide only suggestive outcomes. Therefore, in vitro models including animal (Figure 2A) and human ones (Figure 2B) are used to investigate molecular mechanisms underlying the obesogenic action of EDCs prior to in vivo studies. The first group of in vitro models utilizes preadipocytes and mature adipocytes of humans and different animals such as rodents, although porcine or feline cells have also been used to a lesser extent. The second group of in vitro models is used to study the biotransformation of xenobiotics, assess their toxicity and identify the molecular mechanisms that lead to the disruption of the endocrine function of the crucial metabolic organs that control glucose and lipid homeostasis (e.g., the liver and pancreas).

The main advantage of animal in vitro models over in vivo research is the speed of the experiments, tight control of the environment, reduced cost, well-established protocols, higher throughput and reduced animal use [88]. Regarding adipogenesis, most of these models utilize mouse 3T3-L1 cells to elucidate the mechanisms of EDCs’ action during the preadipocyte differentiation processes. However, it is still unclear if the rodent’s in vitro models are suitable for studying adipogenic responses due to the maintained species specificity, which may have varying responses to obesogens and limit the application of results for human-based risk assessments [89]. Nonetheless, numerous human in vitro models including primary cells from different organs and tissues have been developed and are used to identify obesogens and elucidate the mechanisms of their action implicated in the adipogenic differentiation process, adipose function and hepatotoxicity [8,83,84,90]. Regarding the adipogenesis process, in recent years, mesenchymal stem cells (MSCs) derived from adipose tissue have been utilized as an alternative to animal and human preadipocyte cell models to investigate the mechanisms of obesogenic EDC action, particularly related to disrupting the programming of adipogenesis during a prenatal period, in combination with a Western diet, which leads to a higher risk of obesity in early life and adolescence [91].

## 4. Adipose Tissue Cell Models

### 4.1. Animal Preadipocytes

#### 4.1.1. 3T3-L1 Cell Line

The 3T3-L1 cell line is a well-established in vitro system of white preadipocytes from murine Swiss 3T3 cells [92] and consists of unipotent preadipocytes, which can differentiate only into mature adipocytes [93]. Initiation of the adipogenesis in 3T3-L1 cells requires the treatment of several pro-differentiation agents after cell growth arrest, such as dexamethasone (DEX), insulin, and phosphodiesterase inhibitor 1-methyl-3-isobutyl xanthine (IBMX) [92]. The 3T3-L1 cells are easy to culture and tolerate a very large amount of passages [94]. An important feature of 3T3-L1 cells is their ability to differentiate into both white and brown adipocytes [95]. Unfortunately, 3T3-L1 cells differ between batches from different vendors, which makes them impossible to define as a universal test system [39]. Nonetheless, the 3T3-L1 cell line has been used widely to investigate the effects of various EDCs to establish the molecular mechanisms of adipogenesis and evaluate the potential effects on the risk of obesity [93]. 3T3-L1 cells have been used by Sun et al. [93] to evaluate the molecular mechanisms of a widely used surfactant, 4-hexylphenol (4-HP), as a potential EDC-impaired adipogenesis process. Results showed that 4-HP induced adipogenic differentiation via increasing the mRNA level of PPARγ and its target genes such as fatty acid-binding protein 4 (FABP4) (also known as adipocyte protein 2 (AP2)), fatty acid translocase (CD36), perilipin, and adiponectin, but did not disturb C/EBPα expression. Moreover, in 3T3-L1 cells exposed to 4-HP, a significant increase in lipid accumulation was observed [93]. Choi et al. [7] proved that exposure of 3T3-L1 cells to BPA and its analogous such as bisphenol S (BPS), and bisphenol F (BPF) resulted in increased both mRNA and protein levels of PPARγ, C/EBPα, and AP2. De Filippis et al. [96] reported that BPA had no impact on the PPARγ, FABP4, and FASN expression and adipocyte differentiation but increased mRNA levels of pro-inflammatory markers, tumor necrosis factor-α (TNFα) and interleukin 6 (IL-6) in 3T3-L1 cells. Moreover, Sargis et al. [97] showed that induction of the adipocyte differentiation in the 3T3-L1 cells was promoted by exposure to BPA, dicyclohexyl phthalate (DCHP), and tolylfluanid (TF) through stimulating glucocorticoid receptors (GR), without any significant activation of PPARγ expression. Meruvu et al. [98] evaluated the potential of benzyl butyl phthalate (BBP) on the epigenetic modification of genes involved in adipogenesis. BBP exposure induced miR-34a-5p expression and significantly decreased the expression level of its target genes, including nicotinamide phosphoribosyltransferase (NAMPT), sirtuin 1 (SIRT1), and sirtuin 3 (SIRT3), leading to an impairment in 3T3-L1 preadipocyte differentiation and an increase in adipogenesis [98]. Numerous studies using the 3T3-L1 model have confirmed the obesogenic potential of extensively used different classes of pesticides including quizalofop-p-ethyl (QpE) [99], dichlorodiphenyltrichloroethane (DDT) and dichlorodiphenyldichloroethylene (DDE) [100], diazinon [101], chlorpyrifos (CPF) [102], and tributyltin (TBT) [103], as well as zoxamide, spirodiclofen, flusilazole and acetamiprid [104]. For example, Mangum et al. [50] showed that exposure to 1,1-dichloro-2,2-bis(4-chlorophenyl)ethane (p,p’-DDE) at both the 10 and 20 μM concentrations increased intracellular lipid accumulation by 42% and 58% respectively, compared to the control. The induction of 3T3-L1 preadipocyte differentiation into mature, lipid-storing adipocytes after exposure to these agrochemicals was primarily regulated via PPARγ activation; nonetheless, multiple other obesogenic mechanisms including mitochondrial dysfunction or altered intracellular calcium levels have been also reported [29,105,106,107].

#### 4.1.2. NIH3T3-L1 Cell Line

The NIH3T3-L1 cell line was derived from desegregated NIH Swiss mouse embryo fibroblasts [108]. These cells are adherent, exhibit many physiological similarities to primary adipocytes, and are relatively easy to culture, therefore they are a good model to study adipogenesis and adipocyte function [80,108]. Regarding the EDC exposure, Riu et al. [109] evaluated the effect of tetrabromobisphenol (TBBPA) and tetrachlorobisphenol A (TCBPA), the brominated analogues of BPA, on preadipocyte differentiation and showed that TBBPA and TCBPA, via PPARγ activation, promoted triglyceride accumulation in the NIH3T3-L1 cells. In turn, Howell et al. [80] showed that exposure to DDE did not affect adipogenesis in NIH3T3-L1 cells, but significantly increased the level of adipokines such as resistin, adiponectin, and leptin in mature adipocytes.

#### 4.1.3. 3T3-F442A Cell Line

The 3T3-F442A cell line contains murine preadipocytes of WAT [81,110] which does not require stimulation with DEX and IBMX to differentiate into mature adipocytes [111]. The 3T3-F442A cell line was derived from fibroblasts isolated from disaggregated Swiss mouse embryos and is used to investigate the mechanism of adipogenesis [110,111]. This cell line was applied to investigate the adipogenic potential of DDT, belonging to the group of organochlorine (OC) insecticides that were used heavily during the 1950s and 1960s [112,113,114]. Several epidemiological studies have linked DDT exposure to T2D and obesity [115,116,117]. An in vitro study by Moreno-Aliaga and Matsumura [111] showed that 1,1,1-trichloro-2,2-bis (p-chlorophenyl)-ethane (p,p’-DDT) at a concentration of 20 μM caused 3T3-F442A cells to obtain the adipocyte-like morphology at day 2 of the differentiation process, but treated cells did not fully differentiate. The mouse 3T3-F442A cell line was also used to investigate the impact of BPA exposure on glucose transport [81] and demonstrated that BPA enhanced basal and insulin-stimulated glucose uptake partially via increased GLUT4 protein levels.

#### 4.1.4. OP9 Cell Line

The murine OP9 is an adipocyte cell culture model established from the calvaria of newborn mice genetically deficient in functional macrophage colony-stimulating factor (M-CSF) [94,118]. OP9 cells are bone marrow-derived stromal preadipocytes of WAT characterized by fast adipogenic differentiation and the rapid accumulation of triacylglycerides in lipid droplets after only 72 h of adipogenic stimuli [39,94]. Moreover, OP9 cells can differentiate into adipocytes after reaching confluence and can be passaged for long periods in culture [39]. In comparison with 3T3-L1 cells, OP9 cells are more sensitive to the induction of adipogenesis by chemicals with the ability to activate PPARγ and RXR [19]. OP9 differentiation is a PPARγ-dependent process, and differentiating preadipocytes express C/EBPA, C/EBPβ, as well as perilipin-1 (PLIN1) and perilipin-4 (PLIN4), similar to other adipocyte models [119]. In the last years, the OP9 cell line has been used to evaluate the effects of various compounds on adipogenesis [39,120]. This cell line was used by Kassotis et al. [39] to evaluate the effect of TBT exposure on preadipocyte differentiation. Authors reported that TBT stimulated preadipocyte differentiation, and significantly enhanced triglyceride accumulation in cells on day 7 of differentiation at 100 nM concentration [39]. In turn, 36 potentially adipogenic chemicals identified by the Toxicological Priority Index (ToxPi) on preadipocyte differentiation and adipocyte metabolism in the OP9 cells were also reported by Andrews et al. [120]. Results showed that TBBPA significantly enhanced adipocyte differentiation and had high efficiency in inducing lipid accumulation in OP9 cells at 20 µM [120].

#### 4.1.5. ST-13 Cell Line

The ST-13 cell line was derived from newborn mouse skin and consists of preadipocytes of WAT [82]. Differentiation of the ST-13 preadipocytes is stimulated via ciglitazone and during cell differentiation, the expression of the adipogenic markers such as PPARγ, C/EBPβ and AP2 is induced [121]. The ST-13 cell line has been used by Yamasaki et al. [82] to evaluate the effect of TBBPA exposure on the expression level of genes related to lipid metabolism in differentiated and undifferentiated adipocytes. Exposure to TBBPA at concentrations of 0.5 µM and 1 µM did not have any effects on lipid accumulation and mRNA level of the acetoacetyl-CoA synthetase (AACS) and succinyl-CoA-3-oxoacid CoA-transferase (SCOT) in mature adipocyte cell culture. However, a significantly increased gene expression of lipid and ketone body-utilizing factors such as AACS, PLIN1 and fatty acid synthase (FAS) in both 0.5 µM and 1 µM of TBBPA in ST-13 preadipocytes was noticed. Surprisingly, data showed that the BAT-related factors, uncoupling protein-1 and -3 (UCP-1 and UCP-3), PR domain-containing 16 (PRDM16), lysine-specific demethylase-1 (LSD-1), as well as cell death-inducing DNA fragmentation factor-alpha-like effector A (CIDEA), were overexpressed in ST-13 preadipocytes upon TBBPA treatment [82].

#### 4.1.6. UCP-1 Cell Line

Immortalized UCP-1 reporter brown preadipocytes were generated from Ucp1-luciferase reporter mice and were used to determine the promoter activity of the UCP1 gene [29]. Only one study investigated the effect of EDC exposure on the expression of UCP-1 in immortalized brown adipocytes [29]. Wang et al. [29] investigated the effects of 34 chemicals commonly found in food due to food processing, packaging, and agriculture practices, and showed that only the organophosphate insecticide CPF suppressed UCP1 expression and mitochondrial respiration in BAT at a concentration of 1 pM. Moreover, RNA sequencing showed that at 1 pM CPF, after 4 h exposure, the mRNA levels of the carnitine palmitoyltransferase I A (CPT1A), carnitine palmitoyltransferase I B (CPT1B), and acetyl-coenzyme A acetyltransferase 3 (ACAT3) genes, important for regulating fatty acid oxidation, as well as the cytochrome C oxidase assembly factor (COX16) gene, were reduced [29].

### 4.2. Human Preadipocytes

#### 4.2.1. Primary Human Preadipocytes

Primary human preadipocytes are often applied as in vitro models for the study of preadipocyte differentiation and adipocyte metabolism [122]. They are isolated from adipocyte tissue from different anatomical sites and different donors. Therefore, they reflect donor- and depot-specific characteristics which may lead to some unpredictable differences during experimental studies [94,122,123]. Even though they reflect the characteristics of the donor, they are useful in studies assessing differences between individuals (e.g., obesity, weight loss, age) [94].

Regarding the EDCs, primary subcutaneous human preadipocytes from healthy donors with body mass indices BMI ≤ 24.99 kg m^−2^ were used to investigate the mechanism of BPA-induced adipogenesis [75]. The study showed that BPA exposition increased the expression of C/EBPα and β, PPARγ as well as preadipocyte lipid accumulation in the absence of GR and GR agonist [75]. In another study [70], in vitro microarray analysis showed that human subcutaneous preadipocytes from donors with BMI ≤ 24.99 kg m^−2^ exposed to 50 µM BPA revealed 373 differentially expressed genes, with 235 of those upregulated and 138 genes downregulated. Several genes involved in triglyceride (TG) and lipid metabolism were upregulated after BPA exposure, including acetyl-CoA carboxylase α (ACACA), apolipoprotein A1-binding protein (APOA1BP), perilipin 2 (PLIN2), fatty acid desaturase 1 (FADS1), Niemann-Pick 2 (NPC2), and phosphatidic acid phosphatase type 2A (PPAP2A). In addition, for BPA-treated cells, an increase in mRNA levels was noticed for genes related to lipid metabolism such as sterol regulatory element-binding transcription factor 1 (SREBF1), low-density lipoprotein receptor (LDLR), lipoprotein lipase (LPL), and insulin-induced gene 1 (INSIG1), as well as for those related to adipogenesis, such as growth differentiation factor 15 (GDF15). Moreover, network interaction analysis identified the mammalian target of rapamycin (mTOR) signaling and the thyroid-receptor/retinoid X receptor (TR/RXR) activation as potentially involved in BPA-mediated adipogenesis [70]. Wang et al. [76] analyzed the effect of BPA on human visceral preadipocytes and adipocytes and showed that BPA in the lowest concentration tested (10 nM) increased the mRNA level of 11β-hydroxysteroid dehydrogenase type 1 (11β-HSD1) gene (encoding an enzyme essential in adipogenesis and lipid synthesis). In addition, an increase in PPARγ and lipoprotein lipase (LPL) mRNA levels in preadipocytes and adipocytes was also observed [76]. Currently, scientists are very interested in the molecular effects through which EDCs can disrupt adipose tissue function in children, leading to a risk of childhood obesity and related metabolic syndrome [124]. Therefore, the detrimental effect of BPA on adipogenesis in primary preadipocytes derived from children and modulating endocrine functions has been reported [124]. Menale et al. [124] investigated the molecular mechanisms by which environmentally relevant doses of BPA affect adipogenesis in preadipocytes derived from subcutaneous adipose tissue of non-obese children between 7 and 10 years old. BPA increased the expression of FABP4 and CD36, which are important for lipid metabolism, as well as the expression of proinflammatory cytokines, such as interleukin 1 beta (IL1B), interleukin 18 (IL18) and chemokine (C-C motif) ligand 20 (CCL20) [124].

#### 4.2.2. PCS-210-010

PCS-210-010 cells are commercially available cells containing normal primary subcutaneous human preadipocytes derived from WAT after liposuction surgery. PCS-210-010 cells can proliferate in an undifferentiated state and possess a higher efficiency of adipogenesis than mesenchymal stem cells. Interestingly, PCS-210-010 preadipocytes can be differentiated down osteogenic and chondrogenic lineages [125]. Regarding the EDCs, only one study reported by El-Atta et al. [83] showed that BPA exposure induced the PPARγ, AP2, and peptidylprolyl isomerase A (PPIA) expression as well as increased adiponectin levels in PCS-210-010 cells.

#### 4.2.3. SGBS

SGBS cells were isolated from the stromal vascular fraction of adipose tissue of a male infant with Simpson-Golabi-Behemel syndrome (SGBS) by Wabitsch et al. [122,126]. The SGBS cells exhibit a high capacity for adipose differentiation and were applied in a study of human adipocyte development and metabolism [126]. SGBS cells, unlike primary preadipocytes from healthy donors, retain the ability to differentiate over at least 50 generations [122].

Recently, several studies applied SGBS as a model to investigate the action of a variety of plasticizers such as BPA and phthalates on human adipocyte metabolism [8,84]. For example, Schaffert et al. [8] reported that BPA and its analogues such as BPS, BPF, bisphenol B (BPB) and bisphenol AF (BPAF) displayed significant binding to PPARγ during adipocyte differentiation, but not activated PPARγ at concentrations of 10 nM, 100 nM, 1 µM and 10 µM in SGBS cells. Interestingly, during the differentiation of SGBS preadipocytes, all bisphenols decreased lipid accumulation and BPS, BPB, BPF and BPAF decreased adiponectin levels. Moreover, 1 µM BPA, BPB and BPS considerably reduced insulin sensitivity in SGBS cells upon insulin stimulation [8]. On the other hand, Schaedlich et al. [84] reported that DEHP downregulated the FABP4, adipose triglyceride lipase (ATGL), LPL, lipase E (LIPE), and CD36 mRNA levels, as well as reduced TGs accumulation in lipid droplets of the SGBS cells. Moreover, decreased adiponectin levels and increased leptin secretion in SGBS cells were also observed [84]. Recently, the effects of 20 alternative plasticizers and their metabolites on SGBS preadipocyte differentiation, induction of adipogenic markers and lipid accumulation in mature adipocytes have been reported by Schaffert et al. [62]. The molecules 1,2-cyclohexanedicarboxylic acid mono 4-methyloctyl ester (MINCH), monohydroxy isononyl phthalate (MHINP) and 6-hydroxy monopropylheptyl phthalate (OH-MPHP), which are the metabolites of bis(7-methyloctyl) cyclohexane-1,2-dicarboxylate (DINCH), diisononyl phthalate (DINP), and bis(2-propylheptyl) phthalate (DPHP), respectively, exhibited the highest adipogenic potential by induction of the SGBS preadipocyte differentiation mediated by PPARγ binding and activation. In mature adipocytes, DINCH, DINP and DPHP as well as their metabolites induced oxidative stress and mitochondrial dysfunction, and disturbed lipid storage and adipokine secretion, which was linked to inflammation and insulin resistance [62].

#### 4.2.4. SW 872 Cell Line

SW 872 is a human liposarcoma cell line that differentiates without the differentiation cocktail and they constitutively express PPARγ and C/EBPα, which are crucial to adipocyte development [127,128]. Campioli et al. [129] showed that exposure to MEHP at a concentration of 10 µM activated the SW 872 preadipocyte differentiation and increased the expression of the glucose transporter type 1 and 4 (GLUT1 and GLUT4), calcium-binding protein B (S100B), as well as adenosine triphosphate citrate lyase (ACLY) and ACACA, involved in de novo lipogenesis. Additionally, MEHP temporarily increased the translocator protein (TSPO) mRNA levels during SW 872 adipocyte differentiation [129].

The obesogenic activity of selected EDCs confirmed on adipose tissue cell models is summarized in Table 1.

## 5. Mesenchymal Stem Cells (MSCs)

Mesenchymal stem cells (MSCs) are multipotent and can differentiate into adipocytes, osteocytes, myocytes and chondrocytes [78,93,103,130]. In a variety of studies, MSCs were isolated from bone marrow, adipose tissue, periosteum, muscle tissue, blood vessels, blood, lymphoid organs, lung, skin and umbilical cord [131]. MSCs’ differentiation is controlled via several transcription factors such as octamer-binding transcription factor 4 (OCT4), SRY-box 2 (SOX2) and Nanog homeobox (NANOG) that are responsible for maintaining cells in an undifferentiated state [77]. In addition, MSCs adhere to plastic and express specific surface antigens such as CD19-, CD34-, CD45-, CD79-, CD14 or CD11b-, CD73+, CD90+, and HLA-DR [132]. MSCs are used to study the modulation of stem cell fate under environmental and nutritional factors, and various aspects of adipogenesis in vitro [93,103,129,130].

### 5.1. Animal Adipose-Derived Stem Cells (ADSCs)

The C3H10T1/2 cell line was established in 1973 and derived from C3H mouse embryos that were 14–17 days old [133,134]. C3H10T1/2 cells have a fibroblastic morphology and the capacity to differentiate into adipocytes, chondrocytes and osteocytes [133]. In recent years, C3H10T1/2 cells have been used to investigate the impact of various compounds on preadipocyte differentiation to investigate the molecular mechanisms associated with obesity [94,135,136]. Regarding the EDCs, the C3H10T1/2 cell line was applied to study the impact of parabens such as butylparaben on the disruption of the adipogenesis process. Results showed that exposure to butylparaben stimulated adipogenic differentiation via increased expression of PPARγ, C/EBPα and FABP4, as well as via decreased runt-related transcription factor 2 (RUNX2) mRNA levels, which plays an inhibitory role during adipocyte differentiation [130]. Other studies revealed that BBP induced adipocyte differentiation in C3H10T1/2 stem cells [137,138]. In turn, Zhang and Choudhury [138] showed that expression of the PPARγ and aP2 were significantly increased in C3H10T1/2 stem cells exposed to 50 µM BBP after 8 days of incubation. In addition, decreased SIRT1 mRNA levels, as well as increased β-catenin and forkhead box protein O1 (FoxO1) acetylation under BBP exposure, were also associated with increased adipogenesis [138]. Moreover, the same authors [137] reported that 50 µM BBP significantly downregulated the expression of long non-coding H19 RNA and increased the expression of miR-103/107/let-7 (a, b, c, d, f and g) on day 2 of C3H10T1/2 cell differentiation, which probably stimulated adipogenesis. An adipogenic effect of DEHP, BPA, and TBT as a single compound was also observed in C3H10T1/2 cells by Biemann et al. [139]. Moreover, Biemann, Fisher and Navarrete Santos [140] studied the effects of the EDC mixture at high concentrations (10 µM BPA, 100 µM DEHP, 100 nM TBT), and at environmentally relevant concentrations (10 nM BPA, 100 nM DEHP, 1 nM TBT) and demonstrated that the EDC mixture affects adipogenic differentiation of the C3H10T1/2 cells, but its impact on adipogenesis was dose-dependent. Moreover, in a previous study, Kirchner et al. [78] provided evidence that TBT induced PPARγ2, FABP4 and leptin (LEP) expression in mouse ADSCs while the mRNA level of the adipocyte differentiation-associated protein (PREF-1), an inhibitor of adipocyte differentiation, was decreased.

In recent years ADSCs from domestic animals have gained increased attention because they are a much better model for understanding adipogenesis in vitro and obesity-related diseases compared to rodent cell models [141]. For example, ADSCs isolated from porcine adipose tissue have been applied to study the obesogenic activity of EDCs. Gigante et al. [142] demonstrated that glyphosate (GLY) at the concentration of 4 μg/mL significantly decreased the viability of ADSCs and inhibited their adipogenic differentiation. Similar results were also observed by Berni et al. [143] who noticed a significantly decreased cell viability of proliferating porcine ADSCs after 48 and 72 h of 1 µM BPS exposure. Moreover, similar to GLY, BPS did not increase the PPARγ and LEP expression during the differentiation nor fat droplet formation in porcine ADSCs [143].

### 5.2. Human Adipose-Derived Stem Cells (hADSCs)

Human adipose-derived stem cells (hADSCs) are isolated from biopsies and liposuction specimens [144,145]. hADSCs are available from normal donors and patients with obesity (BMI > 30), Type 1 diabetes or Type 2 diabetes. Moreover, hADSCs have functional and phenotypic characteristics similar to bone marrow-derived mesenchymal stem cells (BMMSC) [145]. Normal hADSCs can differentiate into many different lineages including adipogenic, neural, osteogenic, and chondrogenic cells and can be used in research, including in stem cell differentiation [145]. An important feature of hADSCs is that they can be cultivated up to passage eight with no sign of decline [141]. hADSCs can be used as an alternative to human preadipocytes which have reduced proliferative ability and can exhibit physiological differences related to the fat depot of origin within the body [77]. A main advantage of hADSCs is the commitment of stem cells to preadipocytes and their differentiation to mature adipocytes [146]. hADSCs have been used to assess possible metabolic disruptors in vitro [77,78,147].

Regarding obesogenic EDCs, Valentino et al. [147] showed that the adipose tissue-derived stromal vascular fraction (SVF) exposed to 1 nM BPA exposure decreased insulin-stimulated glucose utilization and increased cytokine secretion such as IL6 and interferon-gamma (IFN-γ). However, no changes in mRNA levels of the adipogenic markers such as GLUT4 and PPARγ were found [147]. Ohlstein et al. [148] showed that BPA enhanced adipogenesis in human ADSCs obtained from subcutaneous abdominal tissue of three female donors with a BMI less than 25. BPA increased the expression of PPARγ, C/EBPα, LPL, insulin-like growth factor-1 (IGF1) and dual leucine zipper-bearing kinase (DLK). It has been reported that the adipogenic effect of BPA was compounded via ER activation, and this effect can be blocked by the ER antagonist ICI 182,780 [148]. De Filippis et al. [96] showed that exposure to 1 nM and 3 nM BPA did not affect the cellular commitment of hADSCs to the adipose lineage, nor did it affect PPARγ, C/EBPα and FABP4 expression or lipid accumulation. Recently, Cohen et al. [55] compared the effects of BPA and BPA replacements such as BPAF and tetramethyl bisphenol F (TMBPF) on adipogenesis and lipid accumulation in female hADSCs. BPA at 0.1 μM and BPAF at 0.1 nM increased adipogenesis and lipid accumulation but higher amounts of BPA (1 μM) and BPAF (10 nM) significantly decreased adipogenesis. Moreover, higher doses of BPA and BPAF were more toxic than lower doses, leading to an increase in cell apoptosis, thus contributing to a decreased level of adipogenesis and fat accumulation. In addition, TMBPF at a concentration of 0.01 µM and 0.1 µM also significantly lowered adipogenesis. This compound exhibited cytotoxic and anti-adipogenic effects on hADSCs, which resulted in increased levels of apoptosis and reduced lipid production [55]. Similar results were also obtained in the study of Harnett et al. [56] who demonstrated that BPA (1, 10 μM), BPAF (0.0003 µM, 0.003 µM, 0.03 µM, 0.3 µM) and TMBPF (0.01 µM, 0.1 µM, 1 µM, 10 µM, 50 µM) had high cytotoxicities and significantly decreased cell viability, leading to massive apoptosis in hADSCs. Reina-Pérez et al. [79] examined the impact of BPF and BPS on lipid accumulation and adipogenesis in hADSCs and showed that these BPA analogues at concentrations of 10 µM or 25 µM enhanced their capacity to differentiate into adipocytes and accumulate lipid droplets in a dose-dependent manner. Another study [77] showed that exposure to DDE maintained the undifferentiated state of hADSCs. DDE influenced the expression of genes involved in maintaining the pluripotent state of cells and differentiation (SOX2, OCT4, NANOG, peroxisome proliferator-activated receptor gamma, coactivator 1 beta (PPARγC1B)), lipid metabolism (FASN, sterol regulatory element-binding protein 1 (SREBP1), UCP3) and members of an insulin signaling pathway (homo sapiens v-akt murine thymoma viral oncogene homolog 2 (AKT2), insulin receptor (INSR)). In turn, hADSCs exposed to TBT showed increased cell differentiation via activation of PPARγ and downregulation of the PREF-1 expression, as an inhibitor of adipocyte differentiation [78].

The obesogenic activity of selected EDCs confirmed on mesenchymal stem cell models is summarized in Table 2.

## 6. Other In Vitro Models of Adipose Tissue and Mesenchymal Stem Cells That Can Be Used for Studying the Mechanism of Obesogenic Action of EDCs

Several immortalized human white adipocyte cell models such as the telomerase reverse transcriptase white preadipocyte cell line (TERT-hWA) [149], LiSa-2 [150], LS14 [151] and Chub-S7 [152], and brown adipocyte cell models such as TERT-hBA [149] and PAZ6 [153], have been generated. These cells, compared to primary cultures, maintain adipogenic potential over time and passages, and therefore have been used over the past few years to study adipocyte function [154].

TERT-hWA and TERT-hBA were isolated by Markussen et al. [149] upon the immortalization of white and brown stromal-vascular cell fractions from superficial and deep neck adipose tissue from a single donor. These cells maintain a fibroblast-like morphology during propagation and exhibit high proliferation and differentiation up to at least passage 20 [149]. The LiSa-2 cell line was isolated by Wabitsch et al. [150] from a poorly differentiated human pleomorphic liposarcoma and displays a high capacity for terminal adipose differentiation [150]. The LiSa-2 cells accumulate lipids and express adipocyte gene markers such as *PPARγ*, *LPL*, *FASN*, hormone-sensitive lipase (*HSL*), adipocyte most abundant gene transcript-1 (*APM1*), glycerol-3-phosphate-dehydrogenase (*GPD1*) and *GLUT4* [150]. LS14 is an adipocyte cell line that was derived from a metastatic liposarcoma and shares many of the characteristics of primary preadipocytes that undergo terminal differentiation, with the expression of many adipose-associated genes [151]. The Chub-S7 cell line was derived from human subcutaneous primary preadipocytes which were transfected with human papillomavirus E7 oncoprotein and the human telomerase reverse transcriptase (hTERT) [152]. Chub-S7 adipocytes display expression of the adipocyte markers and the capacity to accumulate triglycerides [152]. Chub-S7 has been applied in the study of adipocyte differentiation, adipogenic miRNA regulation [154,155], as well as cellular metabolism [154,156].

PAZ6 is the first available immortalized human BAT cell line isolated from infant brown adipose tissue [153,154] and has been used both in white and brown preadipocyte models [154]. Differentiated PAZ6 adipocytes accumulate lipids and express brown/white markers including *UCP1*, β1, β2, and β3 adrenergic receptors (*β-AR*), adrenergic receptor α2A (*α2A-AR*), *LPL*, *GLUT1* and *GLUT4,* as well as *LEP* [153,154]. Moreover, PAZ6 cells can be passaged for several months without losing their molecular markers and morphological characteristics [153]. Therefore, the above-mentioned cell lines can be potential in vitro models to study the obesogenic action of various EDCs classified as food contaminants [149].

Similarly, mouse white adipose cell lines such as Ob17 (epididymal fat cells), TA (embryo fibroblasts), or PFC6 (stromal-vascular fraction of epididymal fat) are used as practical rodent models for studying adipogenic mechanisms and adipocyte biology [110]. Additionally, mouse brown adipose cell lines such as BFC-1 (stromal-vascular fraction of interscapular of BAT), RBM-Ad (BAT/bone marrow, multipotent stem cell line), HB2 (stromal-vascular fraction of interscapular of BAT from p53-knock-out-mouse), HIB 1B and T37i (BAT/brown fat tumors) from different SV40T-transgenic mice have been established [110]. Moreover, porcine [157], feline [158], fetal and adult ovine preadipocytes [159] are used in adipogenesis research.

## 7. Hepatic Cellular Models

The liver is an important detoxification organ in the body and is responsible for the biotransformation and storage of toxic compounds such as exogenous xenobiotics [160]. More than 90% of orally exposed pollutants absorbed via the stomach and intestine are transported to the liver and removed from the body via biotransformation, catalyzed by UDP-glucuronosyltransferases (UGTs) and cytochromes P450 (CYPs) [161]. The liver plays a crucial role in lipid metabolism such as the synthesis and regulation of blood lipids, de novo lipogenesis, fatty acid oxidation, fatty acid uptake, and triacylglycerol export [68,162]. The excessive lipid storage in the liver may lead to lipotoxicity and NAFLD, which very often accompanies obesity [14,93]. Scientific evidence indicates that EDCs affect liver function and lipid accumulation and induce several metabolic syndromes, including hepatic steatosis and hyperlipidemia, but the mechanisms of their action still need to be explained [28,86,163,164]. Nonetheless, several studies have shown that bisphenols, including BPA, BPS and fluorene-9-bisphenol (BHPF), induce liver toxicity and hepatocyte necrosis at low dosages [165,166,167,168].

### 7.1. Animal Hepatocytes

#### 7.1.1. Animal Primary Hepatocytes

Primary hepatocytes isolated from 5 week-old female CD-1 mice were used to evaluate the hepatic toxicity of BHPF [165]. Results showed that exposure to BHPF at 1 µM and 10 µM concentrations increased the lactate dehydrogenase activity (LDH) in the cell culture medium and reduced the hepatocyte numbers [165]. Cocci et al. [169] using hepatocytes from gilthead seabream (*Sparus aurata* L.) showed that diisodecyl phthalate (DIDP) at low concentrations (0.1 to 1 µM) upregulated genes involved in FA desaturation such as stearoyl-CoA desaturase 1A-1B (*SCD1A*, *SCD1B*) and fatty acid desaturase (*FADS2*), FA β-oxidation (*CPT1A* and *CPT1B*), and FA transport and metabolism (apolipoprotein A-I (*APOA-I*), *FABP*, FA synthesis and uptake (*SREBP1*), as well as TG and phospholipid hydrolysis (hepatic lipase (*Hl*) and *LPL*). In turn, Olsvik and Søfteland [170] reported that exposure to the 10 μM *p,p’*-DDE induced the expression of the markers of endocrine disruption such as Vitellogenin 1 (*VTG1*) and estrogen receptor 1 (*ESR1*) in Atlantic salmon hepatocytes. In addition, metabolomics profiling showed that 100 μM *p,p’*-DDE strongly affected diacylglycerol and sphingolipid metabolism, glucose and bile acid metabolism, as well as amino acid metabolism [170].

#### 7.1.2. FaO Cell Line

The rat hepatoma (FaO) cell line is a well-characterized liver cell line used as an in vitro model of steatosis [171]. FaO cells characterize a low mRNA level of the estrogen receptor β (*ERβ*) and lack the estrogen receptor α (*Erα*) expression compared to rat liver [171]. A study by Grasselli et al. [171] showed that BPA at non-cytotoxic concentrations, 30 and 300 ng/mL, induced lipid droplet accumulation and triglyceride content in FaO cells. Additionally, decreased expression of *PPARα*, *γ*, *β* and *δ*, as well as decreased expression acyl-CoA oxidase (*AOX*) and *CPT1* genes involved in lipid oxidation, was observed. BPA had no effect on the expression of lipogenic genes (*FAS*, glycerol-3-phosphate acyltransferase (*GPAT*). Moreover, it lowered the level of mRNA transcripts of apolipoprotein B (*APOB*) and extracellular triglycerides, which suggests that it may cause changes in lipid secretion [171].

#### 7.1.3. BRL-3A Cell Line

The BRL-3A cell line is an epithelial cell line derived from buffalo rat liver [172]. Cells of this line are capable of division in the absence of serum [172]. Using BRL-3A, Zhang et al. [173] showed that MEHP induced cellular lipid accumulation and fatty acid synthesis through inhibition of the JAK2/STAT5 signaling.

#### 7.1.4. AML12 Cell Line

The alpha mouse liver 12 (AML12) cell line is immortalized and not a tumorous cell line that is a widely used cellular model for the study of liver lipid metabolism [174]. Wu et al. [175] reported that polychlorinated biphenyls-153 (PCB-153) disturbed glucose and lipid metabolism via decreased hepatocyte nuclear factor 1b (HNF1b) expression, elevated reactive oxygen species (ROS) levels, and enhanced nuclear factor kappa-light-chain-enhancer of activated B cells (NF-κB)-mediated inflammation, resulting in an increased accumulation of lipid and inhibition of insulin-stimulated glucose uptake in AML-12 cells. A study by Le et al. [176] showed that chlorinated-organophosphorus flame retardants (OPFRs) such as tris (2-chloroethyl) phosphate (TCEP), tris (2-chloroisopropyl) phosphate (TCPP), tris-(2-chloro-1- (chloromethyl) ethyl) phosphate (TDCPP), triphenyl phosphate (TPhP) and tricresyl phosphate (TCP) increased the cell lipid area by 2.3-, 2.5-, 2.7-, 3.3- and 5.2-fold, respectively.

#### 7.1.5. Hepa1-6 Cell Line

Hepa1-6 is a murine hepatoma obtained from the BW7756 hepatoma tumor that emerged spontaneously in C57L/J mice used as an in vitro clinical model for preclinical immunotherapy studies [177]. Regarding EDCs, Ke et al. [178] using Hepa1-6 hepatocytes investigated the effects of BPA at 0.001 µM, and 0.01 µM concentrations on the mRNA level of DNA methyltransferases and genes involved in lipid metabolism. BPA decreased the expression of DNA methyltransferase 1, 3-α and 3-β (*DNMT1*, *DNMT3a*, *DNMT3b*) but increased the *FASN*, 3-hydroxy-3-methylglutaryl CoA reductase (*HMGCR*), *SREBF1* and *SREBF2* genes [178].

#### 7.1.6. FL83B Cell Line

FL83B is a hepatocyte cell line derived from a liver of a 15–17 day-old fetal mouse [179,180]. FL83B hepatocytes actively synthesize cholesterol and store glycogen [181]. The FL83B cells have been used to study the effects of various compounds on hepatocytes’ function, including fucoxanthin [182], herbal tea extracts [183] and heavy metals such as cadmium (Cd) [184]. FL83B cells were used by Lo et al. [185] who showed that DEHP at different concentrations (125 µM, 250 µM, 500 µM, 1000 µM) injured liver FL83B cells by reducing cell viability, increasing LDH and alanine aminotransferase (ALT) release, as well as increasing cell populations of sub-G1 and S phase in a dose-dependent manner.

#### 7.1.7. RTL-W1 Cell Line

RTL-W1 is the epithelial cell line derived from the liver of 4 year-old male rainbow trout [186,187]. This in vitro model was applied to study the ability of EDCs, namely TBT, terpyridine platinum(II) chloride (TPT), 4-nonylphenol (4-NP), BPA and DEHP, to alter the expression of markers of cellular lipid metabolism leading to steatosis in fish [186]. Results presented by Dimastrogiovanni et al. [186] showed that DEHP and BPA significantly increased the accumulation of lipids in RTL-W1 cells, whereas TBT, 4-NP, BPA and DEHP altered membrane lipids such as phosphatidylcholines (PCs) and plasmalogen PCs. Furthermore, RTL-W1 cells exposed to BPA, TBT, TPT, DEHP and 4-NP altered mRNA levels of ATP-binding cassette transporter A1 (*ABCA1*), *CD36*, fatty acid transport protein 1 (*FATP1*), *FAS*, *LPL*, *PPARα* and *PPARβ* [186].

#### 7.1.8. PLHC-1 and ZFL Cell Line

The fish hepatoma PLHC-1 cell line has been derived from topminnow (*Poeciliopsis lucida*) [188,189] whereas the zebrafish liver cell line (ZFL) has been isolated from zebrafish (*Danio rerio*) [188]. These cells maintain several differentiated cell functions of hepatocytes and have been extensively used to assess the cytotoxicity and changes in gene transcription associated with xenobiotic exposure [188,189]. Regarding the effects of EDCs, Marqueño et al. [190] reported that exposure to BPA, BPF and bisphenol A bis(3-chloro-2-hydroxypropyl) ether (BADGE·2HCl) induced the accumulation of ether-triacylglycerides (ether-TGs) and dihydroceramides in hepatic ZFL cells. Moreover, BPA and BADGE·2HCl increased the level of saturated TGs and lowered the levels of unsaturated TGs. Concentrations of 20 µM BPA and 20 µM BPF led to an increase in the expression of the lipogenic genes such as *SCD* and ELOVL fatty acid elongase 6 (*ELOVL6*), while the *PPARα* mRNA level was down-regulated by 20 µM BPF and 5 µM BADGE·2HCl [190]. In the PLHC-1 cells, exposure to BADGE·2HCl induced a strong decrease of triacylglycerides (TGs), while DEHP and dibutyl phthalate (DBP) stimulated the accumulation of TGs [191]. Furthermore, the effect of TBT on the dysregulation of lipid metabolism in PLHC-1 and ZFL cells, as well as the alteration of the *FASN*, *SCD*, and *ELOVL6* expression in ZFL cells, was also reported [188].

### 7.2. Human Hepatocytes

Since the liver is the main organ involved in the metabolism and the toxicity of xenobiotics, isolated primary human hepatocytes (PHHs) have been increasingly used as a model in pharmaco-toxicological studies for the detection of toxic chemicals and evaluating their mechanism of toxicity [192,193]. While PHHs represent a valuable tool for studying liver function, the main limitation of their utilization is the restricted accessibility, heterogeneity, phenotypic instability and limited time for cell proliferation in in vitro culture [193,194]. Therefore, alternative hepatocyte models have been explored and used, including cells from human liver tumors or immortalized adult or fetal human hepatic cells [193]. The advantage of these cell lines is their unlimited availability and rapid growth, but they are dedifferentiated, and compared to normal adult hepatic cells, they show less liver-specific metabolism [195].

#### 7.2.1. Human Primary Hepatocytes

Primary hepatocytes isolated from cancer-free portions of the liver after resection were used to assess the level of FA accumulation upon 2,3,7,8-tetrachlorodibenzo-p-dioxin (TCDD) exposure [90]. TCDD increased total FAs in hepatocytes, including stearate, palmitate, oleic and linoleic acids after 48 h exposure at a 10 nM concentration [90].

#### 7.2.2. HepG2 Cell Line

HepG2 is the best-characterized human hepatoma cell line [194]. This cell-based model is cost-effective, easy to handle, and ensures the repeatability of the obtained results [194]. Regarding the differences between the HepG2 cell line and normal hepatocytes, HepG2 cells do not possess the complete set of xenobiotic-metabolizing enzymes (XMEs), especially UDP-glucuronosyltransferases (UGTs) and some cytochromes P450 (CYPs) such as CYP2A6, CYP2D6, CYP3A4, CYP2C9, CYP2C19, etc., that are involved during phase I of xenobiotic oxidation in the liver [164,196,197]. Despite this, HepG2 cells retain most of the metabolic functions performed by normal hepatocytes, which allows them to be used in studies of the toxic effects of drugs, nanoparticles, and heavy metals in vitro [198]. The HepG2 cell line has been applied in hepatotoxicity assessments [194] and has been used to evaluate the link between EDC exposure and fatty liver disease associated with an increased risk of obesity [93]. Regarding the surfactants, Sun et al. [93] reported that exposure to 4-HP increased lipid accumulation in oleic acid (OA)-treated HepG2 cells and inhibited de novo lipogenesis by decreasing the acetyl-CoA carboxylase (ACC) and SREBP1c expression as well as the fatty acid oxidation by decreasing the PPARα and CPT1A mRNA levels. Moreover, 4-HP accelerated the uptake process of OA in hepatocytes by an increase of the CD36 mRNA level [93]. In turn, Lu et al. [199] reported that 1,3-dichloro-2-propanol (1,3-DCP) at a 0.5 to 2 µg/mL concentration increased lipid droplet accumulation as well as total cholesterol (TC) and TGs content in HepG2 cells. The molecule 1,3-DCP considerably increased the mRNA level of LDLR, SREBP2 and HMGCR, associated with lipid metabolism [199]. Furthermore, an increase in the lipid accumulation in HepG2 cells exposed to pentabromotoluene (PBT), hexabromocyclododecane (HBCD), and tetrabromobenzoate (TBB) was also reported by Maia et al. [200]. Recently, Vasconcelosa, Silva and Louro [57] showed that DINCH as a non-phthalate plasticizer induced oxidative DNA damage in HepG2 cells, which can be correlated to numerous human diseases including diabetes and cardiovascular disease.

#### 7.2.3. HepaRG Cell Line

HepaRG is an immortalized hepatic cell line that has a similar expression of nuclear receptors, key metabolic enzymes (XMEs), and drug transporters as primary human hepatocytes [201]. HepaRG is a good model used in the field of toxicology because HepaRG cells can enter into a differentiation program toward hepatocyte-like and biliary-like cells [164]. Regarding pesticides, Stossi et al. [202] showed that TBT induced lipid accumulation in HepaRG cells via increased mRNA levels of SREBF1 and FASN involved in de novo lipogenesis. On the other hand, HepaRG cells exposed to BPA had significantly greater cellular triglyceride and neutral lipid accumulation at a 2 nM concentration [164]. BPA induced hepatic lipid accumulation by increasing the apolipoprotein A4 (APOA4) mRNA level, whereas no effect on perilipin 3 (TIP47) and perilipin 2 (PLIN2) gene expression, involved in lipid droplets accumulation, nor for genes associated with carbohydrate homeostasis, was observed [164].

#### 7.2.4. HPR116 Cell Line

HPR116 cells, which are differentiated HepaRG cells, are used to save time in the experiment because they are ready-to-use and easy to use. Cells from the same batch show a repeated differentiation level and have the same behaviour. In addition, HPR116 cells are long living, remaining viable and usable for at least two weeks. They exhibit responses and functions similar to those of primary hepatocytes [203].

#### 7.2.5. Huh-7 Cell Line

Huh-7 (human hepatoma) is an immortalized cell line consisting of tumorigenic cells [204]. Wada et al. [205] showed that BPA and 4-NP exposure stimulated lipid accumulation in Huh-7 cells. Similarly, Lee et al. [206] confirmed that BPA increased intracellular lipid accumulation and fatty acid uptake in Huh-7 cells.

#### 7.2.6. Huh-6 Cell Line

Fetal HuH6 hepatocytes from a hepatoblastoma of a 1 year-old male donor are also used in in vitro tests of the endocrine-disrupting effects of different substances [207,208]. The advantages of this cell line include availability, unlimited growth, high reproducibility of results, and the expression of enzymes involved in the metabolism of xenobiotics [209].

#### 7.2.7. HHL-5 Cell Line

HHL-5 is an immortalized human hepatocyte cell line. Its phenotype resembles primary hepatocytes [210]. Martella et al. [210] demonstrated that exposure of HHL-5 cells to BPA induced FA accumulation in an endocannabinoid receptor type 1 (CB1)-dependent manner. BPA increased CB1 activity by stimulating the synthesis of anandamide (N-arachidonoylethanolamine; AEA) [210].

#### 7.2.8. L02 Cell Line

The human normal liver cell line L02 is used to study the effects of various compounds on lipogenesis [211]. The L02 cell line was derived from primary normal human hepatocytes and immortalized in 1980 [212]. This cell line has found applications in the research of human hepatocellular functions such as drug hepatotoxicity, hepatic steatosis, and chemical carcinogenesis [212]. Zhang et al. [213] showed that triclosan (TCS), widely used as an antibacterial and antifungal agent, promoted the perturbation of intracellular lipids in L02 cells.

The obesogenic effects of selected EDCs confirmed in hepatic cellular models are summarized in Table 3.

## 8. Pancreatic Cellular Models

The α and β cells of the pancreas play an important role in blood glucose control through the secretion of glucagon and insulin [14]. Glucagon secreted by α-cells is involved in the synthesis and mobilization of glucose in the liver [214]. The insulin secreted by β-cells reduces blood glucose levels via increasing glucose uptake by insulin-sensitive tissues, such as the liver, adipose tissue and skeletal muscle, and inhibiting hepatic glucose production [68]. Type 2 diabetes mellitus (T2DM) is characterized by hyperglucagonemia and hypoinsulinemia, which results in increased blood glucose levels [14]. T2DM is comprised of a series of interrelated abnormalities such as insulin resistance (IR) and metabolic syndrome [28]. IR in WAT, skeletal muscle and liver combined with inappropriate insulin secretion from pancreatic β cells is the major cause of human T2DM [68]. Recently, an increasingly significant role in the development of T2DM is attributed to EDCs [215]. Moreover, EDCs may be of great importance in the pathogenesis of Type 1 diabetes, especially during the developmental period [23].

### 8.1. Animal Pancreatic Cells

#### 8.1.1. Rat Pancreatic Islets

Pancreatic islets isolated from male Wistar rats at 8 weeks of age were used to investigate the effects of acute and long-term exposure to BPA and NP on insulin secretion [216]. Adachi et al. [216] showed that acute exposure (60 min) to BPA and NP at 0.1, 1, 10 and 100 µg/L did not affect insulin secretion in pancreatic cells with glucose stimulation. In turn, 24 h exposure to BPA (10 and 100 µg/L) or NP (0.1, 1, 10 and 100 µg/L) with 16.7 mM glucose significantly increased insulin secretion via cytosolic/nuclear estrogen receptors [216]. Ghaemmaleki et al. [217] reported that 10 µM TBT reduced the viability of the pancreatic islets of Langerhans isolated from 2–3 month-old male Wistar rats by 50%. In addition, increased insulin secretion at both basal (2.8 mM) and stimulatory (16.7 mM) concentrations of glucose after 10 µM TBT exposure was observed [217].

#### 8.1.2. INS-1 Cell Line

The INS-1 cells were isolated from a rat insulinoma induced by X-ray irradiation and are applied in the studies of insulin secretory mechanisms. INS-1 cells are bi-hormonal and are capable of expressing both insulin and proglucagon proteins [218]. There is strong evidence that BPA in the concentration range from 0.002 to 2 µM lowers the viability of INS-1 cells and increases apoptosis via a mitochondria-mediated pathway in a dose-dependent manner [219]. Interestingly, BPA at higher concentrations (0.2 and 2 µM) significantly decreased insulin secretion in response to glucose stimulation, but at 0.002 µM slightly increased insulin secretion [219].

#### 8.1.3. INS-1E Cell Line

The INS-1E cell line is a stable rat insulinoma pancreatic β-cell line cloned from the INS-1 cells, characterized as less heterogeneous than the INS-1 cell line [23]. INS-1E cells are widely used in studies of β cell function [23]. It has been reported that BPA (1 µM) and TBT (200 nM) decreased INS-1E cell viability by inducing apoptosis [23]. In addition, TBT caused a reduction in the expression of the MAFA, which is a transcription factor regulating the expression of genes involved in the biosynthesis and secretion of insulin (such as pancreatic and duodenal homeobox 1 (PDX1) and glucokinase (GCK)), as well as Pdx1. Moreover, triphenylphosphate (TPP), PFOA (perfluorooctanoic acid), TCS and DDE at a concentration of up to 1 µM did not affect the viability of INS-1E cells, nor the expression level of genes involved in insulin biosynthesis and secretion [23]. Another study showed that p,p’-DDT (10 µM) and its metabolite p,p’-DDE (10 µM) reduced the intracellular level of proinsulin (precursor of insulin) and insulin monomer (the active form of insulin), as well as decreased insulin 1 (INS1) and 2 (INS2) mRNA levels in INS-1E cells [220]. Additionally, p,p’-DDT decreased the intracellular level of hexameric insulin, which is a final form of insulin secreted by pancreatic β cells. p,p’-DDT decreased the expression of actin and mortalin/GRP75 and increased the expression of tubulin beta-5 chain, annexin A4, and vitamin D-binding protein (VDBP). In turn, p,p’-DDE also increased VDBP expression but decreased glucosidase 2 subunit beta (GLU2β) precursor expression [220].

#### 8.1.4. RIN-m5F Cell Line

The RIN-m5F cell line is a rat pancreatic β-cell line that can produce and secrete insulin [106,221]. Chen et al. [106] demonstrated that TBT at the concentration between 0.05 and 0.2 µM did not affect the viability of RIN-m5F cells after 24 h incubation, but increased glucose-stimulated insulin secretion (GSIS) in β cells after 0.1 and 0.2 µM TBT treatment. In a study reported by Huang et al. [221], TBT at a 0.5 µM dose increased the number of apoptotic RIN-m5F cells after 24 h incubation, which was associated with the phosphorylation of mitogen-activated protein kinases (MAPKs)-c-Jun N-terminal kinase (JNK), as well as extracellular signal-regulated protein kinase (ERK1/2) and poly (ADP-ribose) polymerase (PARP) cleavage. Furthermore, TBT (0.5 µM, 1 µM) was found to significantly decrease GSIS after 24 h treatment [221]. In turn, Suh et al. [222] reported that PFOA at the 100–500 µM concentration range significantly decreased the viability of RIN-m5F cells and increased their apoptosis. It turned out that this EDC caused oxidative stress and mitochondrial dysfunction via the reduction of adenosine triphosphate (ATP) level, as well as induction of cardiolipin peroxidation, mitochondrial membrane potential collapse as well as cytochrome c release [222].

#### 8.1.5. Mouse Pancreatic Islets

The mouse islets derived from adult male mice (12–14 weeks old) have been used by Dos Santos et al. [23] who demonstrated that BPA (1 nM, 1 µM) and TBT (20 nM, 200 nM) promoted apoptosis in dispersed mice islets. Carchia et al. [105] showed that BPA at a low dose (0.001 µM) changed the functioning of primary murine pancreatic islets and glucose homeostasis. BPA led to the dysfunction of the mitochondria and their destruction by inhibiting the expression of genes important in mitochondrial activity. This caused a decrease in insulin secretion by β cells after 1 h glucose stimulation (16 mM), as well as a decrease in the viability of these cells [105]. Soriano et al. [223] reported that BPA at a concentration of 1 nM increased insulin secretion and decreased ATP-sensitive K^+^ (K_ATP_) channel activity in β cells from wild-type (WT) mice (C57), which was not recorded in cells from estrogen receptor β (ERβ) knockout (ERβ -/-) mice (BERKO mice). Recently, Marroqui et al. [224] showed that BPS and BPF at 1 nM and 1 µM concentrations increased insulin secretion and lowered K_ATP_ channel activity (1 nM BPS, 10 nM BPF) in pancreatic β cells from WT mice (C57BL/6J). In turn, Chen et al. [106] reported that 0.1 µM and 0.2 µM TBT increased GSIS in isolated mouse islets.

#### 8.1.6. MIN-6 Cell Line

The MIN6 cell line is derived from a mouse insulinoma. This transformed β-cell line retains GSIS and is used to study insulin secretion [225,226]. Nonetheless, it should be noted that the long-term culture of MIN6 cells results in the loss of their insulin secretory capacity in response to glucose [227]. This is probably because β-cells dominating in the culture respond poorly to glucose or due to the increased expression of genes responsible for GSIS changes over time [228]. Al-Abdulla et al. [229] showed that 100 nM BPA increased GSIS. Moreover, upregulation of, e.g., MAFA, hepatocyte nuclear factor 4 alpha (HNF4α) and PDX1, which are important for insulin secretion and normal glucose sensing, was observed in cells treated with BPA. In turn, BPS, DEHP, perfluorooctanesulfonic acid (PFOS) and DDE decreased insulin release, while cadmium chloride (CdCl_2_) had no effect on GSIS in MIN-6 cells [229].

#### 8.1.7. β-TC-6 Cell Line

The beta-tumor cell-6 (β-TC-6) cell line is a mouse islet β-cancer cell line derived from a transgenic mouse expressing genes encoding insulin, glucagon and somatostatin; β-TC-6 cells are capable of secreting insulin in response to glucose [230]. The β-TC-6 cell line was applied in the study reported by Qin et al. [231] who showed that exposure to 50 µM and 100 µM PFOS stimulated GSIS in β-TC-6 cells and increased intracellular calcium levels via G protein-coupled receptor 40 (GPR40) activation. In another study [232], 10 µM p,p’-DDE significantly increased basal and GSIS in β-TC-6 cells. Scientists speculated that this EDC does not affect insulin transcription because it does not increase the levels of PDX1 that regulates insulin gene transcription. Probably, DDE alters insulin translation by increasing the level of prohormone convertase (PC), which is involved in the cleavage of insulin to its mature form [232].

The literature also describes other animal insulin-secreting cell lines used in diabetes mellitus research such as murine cell lines (β-TC-1, β-TC-3, IgSC195, βHC, NIT-1), rat cell lines (RINm, RINr, BRIN-BG5, BRIN-BG7, BRIN-BD11, CRI-G1, CRI-G1-RS, In-111) and hamster pancreatic β-cell lines (HIT-T15) [227,233], but to the best of our knowledge, the effect of EDC exposure on the development of obesity and metabolic disorders using these in vitro models has not yet been investigated.

### 8.2. Human Pancreatic Cells

#### 8.2.1. Human Pancreatic Islets

Studies conducted on β-cells from the islets of Langerhans from different human donors demonstrated that 1 nM BPA decreased K_ATP_ channel activity (closure of K_ATP_ channels), which contributed to an increase in GSIS [223]. Moreover, Chen et al. [106] reported that 0.1 µM TBT significantly increased GSIS in human islets from patients with benign pancreatic tumors.

#### 8.2.2. EndoC-βH1 Cell Line

The EndoC-βH1 is a human cell line widely used in diabetes and islet biology research [23]. These cells express all genes that determine the primary β-cells phenotype. However, unlike primary cells, EndoC-βH1 cells may show a different expression of disallowed genes and some β-cell markers and contain approximately 5–10% of the insulin present in native β-cells [23]. Moreover, EndoC-βH1 cells have a greater ability to proliferate than adult human β-cells and present similar insulin secretion in response to glucose in human islets [23].

Using EndoC-βH1cells with BPA and TBT as positive controls, Dos Santos et al. [23] evaluated the adverse effects of PFOA, TPP, TCS, and DDE exposure on β-cell viability and GSIS. Results showed that 1 µM of the DDE, TCS, TPP, and PFOA did not affect the viability, whereas higher concentrations of PFOA (20 to 200 µM) induced apoptosis in the β-cells upon 24 h treatment. In contrast, 1 µM BPA and 200 nM TBT reduced the cell viability and induced the apoptosis of the β-cells. BPA and TCS did not affect GSIS whereas TPP, DDE, and TBT increased insulin secretion. Interestingly, PFOA decreased insulin secretion both at high and low glucose concentrations. All tested compounds, except TBT, did not modify the insulin content. PFOA, BPA, TCS, TPP, and DDE did not affect the expression of genes related to insulin biosynthesis and secretion in comparison to TBT, which increased the glucose transporter type 2 (GLUT2) expression in EndoC-βH1 cells [23]. Al-Abdulla et al. [229] investigated the effects of BPA, BPS, BPF, PFOS, DEHP, CdCl_2_ and DDE exposure at different concentrations ranging from 100 pM to 10 μM on human pancreatic β-cell function. BPA, PFOS and CdCl_2_ treatment resulted in a marked increase in GSIS, whereas a decrease in insulin secretion in EndoC-βH1 cells upon BPS and DEHP exposure was observed. BPF and DDE had no effect on insulin release. Regarding BPS, a significant decrease of the GLUT1, MAFA, MAFB, synaptosome-associated protein 25 (SNAP25) and KIR6.2 mRNA levels in pancreatic β-cells was also noticed [229].

#### 8.2.3. NES2Y Cell Line

The NES2Y is a human pancreatic β-cell line characterized by a constitutive insulin release and possesses an insulin promoter unresponsive to changes in glucose levels [234]. NES2Y cells are proliferative, lack functional ATP-sensitive potassium channels (K_ATP_), and also carry a defect in the insulin gene-regulatory transcription factor (PDX1) [234].

Regarding EDCs, Pavlikova et al. [234] verified that p,p’-DDT and p,p’-DDE at 100 μM concentrations induced a time-dependent inhibition of pancreatic β-cell proliferation and observed that 10 µM p,p’-DDT after 1 month of exposure downregulated the levels of the three cytoskeletal proteins (actin (ACTB), cytokeratin 18 (CK18) cytokeratin 8 (CK8)) and alpha-enolase (ENO1), involved in glycolysis. In turn, 10 µM of p,p’-DDE decreased the expression of heterogeneous nuclear ribonucleoprotein H1 (HNRH1) and CK18 [234]. Moreover, it has been shown that high concentrations of DDT (150 µM, 175 µM, 200 µM) reduced NES2Y cell viability after 24 h of exposure. In addition, NES2Y cells exposed to 150 μM DDT showed decreased levels of 22 proteins such as mitochondrial proteins (enoyl-CoA hydratase (ECHM), 75 kDa glucose-regulated protein (GRP75), NADH dehydrogenase (ubiquinone) iron-sulfur protein 3 and 1 (NDUS3 and NDUS1), proteins involved in the endoplasmic reticulum (ER) stress (endoplasmin, 78 kDa glucose-regulated protein (GRP78)), proteins associated with maintenance of the cell morphology (T-complex protein 1 subunit alpha (TCPA), ezrin, EF-hand domain-containing protein (D2EFHD2), N-myc downstream regulated 1 (NDRG1)), as well as other proteins such as heat shock protein 27 (HSP27), polysaccharide biosynthesis domain-containing 1 (PBDC1) and proliferating cell nuclear antigen (PCNA) [107].

#### 8.2.4. PANC-1 Cell Line

The PANC-1 cell line is an epithelioid carcinoma cell line derived from the human pancreas [235] and is widely used as a human model of pancreatic cells because of the cells’ ability to secrete insulin in response to high amounts of glucose in the culture medium [236]. Using the PANC-1 line, Menale et al. [124] reported that 10 nM BPA effectively impaired insulin secretion in the exposed cells via downregulation of the proprotein convertase subtilisin/kexin type 1 (PCSK1) expression gene involved in insulin production. Other human pancreatic beta-cell lines such as CM, TRM-1, and Blox5 have also been used in adipogenic differentiation studies [227]. However, to the best of our knowledge, they have not yet been applied to study the impact of EDCs on the adipogenesis process.

The obesogenic effects of selected EDCs on pancreatic cellular models are summarized in Table 4.

## 9. Conclusions

In this review, we have made extensive insight into the available literature regarding animal and human 2D in vitro cell models applied to evaluate the obesogenic action of various environmental EDCs. According to the available literature, animal in vitro cell models especially from rodents are extensively used for assessing adipogenesis and applied to screen environmental obesogens. The main advantage of animal in vitro cell models is their commercial availability and well-established protocols for their cultivation. Nonetheless, the translation from animal models is limited by their metabolic heterogeneity, especially between murine and human types. To reduce this risk, human primary cells isolated from adipocyte tissue and crucial endocrine organs such as the liver and pancreas are increasingly used. The main advantage of these cells is comparable identity and functions to native tissue, but their maintenance under in vitro conditions still remains a challenge, because such cells are characterized by a limited number of divisions, after which they lose their biochemical functionality and undergo programmed cell death. Recently, there has been a growing interest in the derivation of hADSCs that does not require cell transformation. The main advantage of hADSCs is the possibility of studying the exposure of chemical compounds on adipo- and lipo-genesis mechanisms by omitting the extrapolation step.

It should be noted that in vitro models are a relatively simplified system compared to the complexity of a living organism and its response to exogenous factors. It is difficult to estimate what concentration of a given compound in vitro corresponds to an in vivo dose, as well as to analyze interactions between different cell types and simulate the effects of long-term exposure to a given compound in cellular models. In addition, it is also worth noting that in vitro tests can provide unreliable results, e.g., in the case of examining the obesogenic effect of phthalates, as they are present in some laboratory plastics. Moreover, in studies with the use of cell models, not only should individual EDCs be tested, but also EDC mixtures, which reflect the “real-life” obesogenic effect much better.

In summary, in vitro studies carried out mainly on cell cultures or isolated tissue samples are used extensively to investigate the mode of action of possible industrial obesogens. While in vitro models have limitations that must be resolved, they are generally simpler, more cost-effective and can be performed in a large series of experiments under the same conditions. Nonetheless, for a better understanding of the mechanisms of the obesogenic EDCs, all information from in vitro and in vivo models should be combined.

## Figures and Tables

**Table 1:** The obesogenic effects of selected EDCs confirmed on adipose tissue cell models.

Cell Type	Organism	In vitro Model	EDCs	Mechanism of Action	Concentration *	References
**Preadipocytes**	**Animal**	**3T3-L1 cell line**	4-HP	adipogenic differentiation ↑ intracellular lipid accumulation ↑ (10 µM, 20 µM) mRNA level of *PPARγ*, *FABP4*, *CD36*, *perilipin*, *adiponectin* ↑ (10 µM, 20 µM); *C/EBPα* (−) (10 µM, 20 µM)	10 µM, 20 µM	Sun et al. [93]
			BPA	lipid accumulation ↑ mRNA and protein levels of PPARγ, C/EBPα, and AP2 ↑	20 µM	Choi et al. [7]
				adipogenic differentiation (–) (1 nM, 10 nM), ↓ (100 nM) mRNA level of *PPARγ*, *FABP4*, *FASN* (–) (1 nM, 10 nM), ↓ (100 nM); *TNFα*, *IL6* ↑ (1 nM, 3 nM) insulin-stimulated glucose uptake ↓ (1 nM)	1 nM, 3 nM, 10 nM, 100 nM	De Filippis et al. [96]
				adipogenic differentiation ↑ lipid accumulation ↑ (100 nM) glucocorticoid-like activity ↑ (1 µM) PPARγ activity ↑ (however, less activation of PPARγ than GR) (1 µM) protein expression of IR-β (insulin receptor subunit β) ↑; C/EBPα ↑ (1 µM–100 pM), adiponectin ↑ (10 nM, 100 nM)	100 pM, 1 nM, 10 nM, 100 nM, 1 µM	Sargis et al. [97]
			BPS	lipid accumulation ↑ mRNA and protein levels of PPARγ, C/EBPα, and AP2 ↑	20 µM	Choi et al. [7]
			BPF	lipid accumulation ↑ mRNA and protein levels of PPARγ, C/EBPα, and AP2 ↑	20 µM	Choi et al. [7]
			TBBPA	Adipogenesis ↑ lipid accumulation ↑ (20 µM) PPARγ activity ↑ (10, 20 µM)	20 µM	Andrews et al. [120]
			BBP	adipogenic differention ↑ lipid accumulation ↑ (1 µM, 10 µM, 50 µM), (–) (0.001 µM, 0.01 µM, 0.1 µM) miR-34a-5p expression level ↑ (1 µM, 10 µM, 50 µM), (−) (0.01 µM, 0.1 µM) mRNA level of *PPARγ* ↑ (10 µM, 50 µM), (–) (0.01 µM, 0.1 µM, 1 µM); *AP2* ↑ (50 µM), (–) (0.01 µM, 0.1 µM, 1 µM); *NAMPT*, *SIRT1*, *SIRT3* ↓ (0.01 µM, 0.1 µM, 1 µM, 10 µM, 50 µM) protein level of NAMPT ↓, SIRT1, SIRT3 (–) (0.1 µM, 50 µM) NAD/NADH ratio ↓ (0.1 µM, 50 µM)	0.01 µM, 0.1 µM, 1 µM, 10 µM, 50 µM	Meruvu et al. [98]
			DCHP	adipogenic differentiation ↑ lipid accumulation ↑ (100 nM) glucocorticoid-like activity ↑ (1 µM) PPARγ activity ↑ (however, less activation of PPARγ than GR) (1 µM) protein expression of IR-β ↑, C/EBPα ↑ (1 µM–100 pM), adiponectin ↑ (1 µM–100 pM)	100 pM, 1 nM, 10 nM, 100 nM, 1 µM	Sargis et al. [97]
			endrin	adipogenic differentiation ↑ lipid accumulation ↑ (100 nM) glucocorticoid-like activity ↑ (1 µM) PPARγ activity ↑ (however, less activation of PPARγ than GR) (1 µM) protein expression of IR-β ↑, C/EBPα ↑ (1 µM–100 pM), adiponectin ↑ (100 nM–100 pM)	100 pM, 1 nM, 10 nM, 100 nM, 1 µM	Sargis et al. [97]
			TF	adipogenic adifferentiation ↑ lipid accumulation ↑ (100 nM) glucocorticoid-like activity ↑ (1 µM) PPARγ activity ↑ (however, less activation of PPARγ than GR) (1 µM) protein expression of IR-β ↑, C/EBPα ↑ (1 µM–100 pM), adiponectin ↑ (1 µM–100 pM)	100 pM, 1 nM, 10 nM, 100 nM, 1 µM	Sargis et al. [97]
			QpE	adipogenesis ↑ lipid accumulation ↑ (5–100 µM) PPARγ activity ↑ (100 µM)	5–100 µM	Biserni et al. [99]
			p,p’-DDT	adipogenesis ↑ lipid accumulation ↑ (20 µM) mRNA level of *C/EBPα*, *PPARγ*, *FAS*, *ACC*, *ATGL*, *HSL*, *LEP* ↑ (10 µM, 20 µM) C/EBPα, PPARγ, AMPKα, ACC protein expression ↑ (10 µM, 20 µM) phosphorylated forms of AMPKα, ACC protein expression ↓ (10 µM, 20 µM)	10 µM, 20 µM	Kim et al [100]
				adipogenesis ↑ lipid accumulation ↑ (10 µM, 20 µM, 30 µM, 50 µM) protein level of C/EBPβ (–), C/EBPα ↑ (20 µM); PPARγ1, PPARγ2 ↑ (10 µM, 20 µM, 30 µM)	10 µM, 20 µM, 30 µM, 50 µM	Moreno-Aliaga and Matsumura [111]
			p,p’-DDE	adipogenesis ↑ lipid accumulation ↑ (10 µM, 20 µM) mRNA level of *C/EBPα*, *PPARγ*, *FAS*, *ACC*, *ATGL*, *HSL*, *LEP* (10 µM, 20 µM), *Lpl* (20 µM) ↑ C/EBPα, PPARγ, AMPKα, Acc protein expression ↑ (10 µM, 20 µM) phosphorylated forms of AMPKα, ACC protein expression ↓ (10 µM, 20 µM)	10 µM, 20 µM	Kim et al. [100]
				adipogenesis ↑ lipid accumulation ↑ (10 µM, 20 µM) mRNA level of *SREBF1*, *FASN*, *PPARγ*, *LEP*, *FABP4* ↑ (2.5 µM, 10 µM, 20 µM)	2.5 µM, 10 µM, 20 µM	Mangum et al. [51]
			diazinon	adipogenesis ↑ lipid accumulation ↑ (1 µM, 10 µM, 25 µM, 50 µM, 100 µM) mRNA level of *C/EBPα*, *PPARγ*, *FASN* ↑ (10 µM) C/EBPα, PPARγ, FASN, ACC, adiponectin, perilipin protein expression ↑ (10 µM, 100 µM)	1 µM, 10 µM, 25 µM, 50 µM, 100 µM	Smith, Yu, Yin [101]
			CPF	adipogenesis ↑ lipid accumulation ↑ (10 µM, 50 µM) mRNA level of *C/EBPα*, *PPARγ*, *FABP4* ↑ (50 µM) protein expression of C/EBPα, PPARγ, FABP4 ↑ (50 µM)	10 µM, 50 µM	Blanco et al. [102]
			TBT	adipogenesis ↑ lipid accumulation ↑ (50 nM) PPARγ activity ↑ (5 nM, 50 nM, 100 nM) basal glucose uptake (50 nM)	5 nM, 50 nM, 100 nM	Regnier et al. [103]
			zoxamide	adipogenesis ↑ lipid accumulation ↑ (0.02 µM) PPARγ activity ↑ (EC_50_ = 1.31 µM, EC_10_ = 0.31 µM) mRNA level of *FABP4* ↑ (2 µM)	0.02 µM, 0.31 µM, 1.31 µM, 2 µM	Janesick et al. [104]
			spirodiclofen	adipogenesis ↑ lipid accumulation ↑ (0.02 µM, 0.2 µM, 2 µM, 10 µM, 20 µM) PPARγ activity ↑ (EC_50_ = 12.76 µM, EC_10_ = 7.27 µM) mRNA level of *LPL* ↑ (0.02 µM, 2 µM, 10 µM, 20 µM)	0.02 µM, 0.2 µM, 2 µM, 7.27 µM, 10 µM, 12.76 µM, 20 µM	Janesick et al. [104]
			flusilazole	adipogenesis ↑ lipid accumulation ↑ (0.02 µM, 0.2 µM, 2 µM) mRNA level of *FABP4* ↑ (0.02 µM), *FSP27* ↑ (0.02 µM, 0.2 µM), *LPL* ↑ (0.02 µM)	0.02 µM, 0.2 µM, 2 µM	Janesick et al. [104]
			acetamiprid	adipogenesis ↑ lipid accumulation ↑ (0.2 µM) mRNA level of *FABP4*, *FSP27*, *LPL* ↑ (0.2 µM)	0.2 µM	Janesick et al. [104]
		**NIH3T3-L1 cell line**	TBBPA	adipogenesis ↑ lipid accumulation ↑ ERα, ERβ, PPARγ activity ↑ mRNA level of *APOA2/FABP4* (*AP2*), *PPARγ* ↑	10 µM	Riu et al. [109]
			TCBPA	adipogenesis ↑ lipid accumulation ↑ ERα, ERβ, PPARγ activity ↑ mRNA level of *APOA2/FABP4* (*AP2*), *PPARγ* ↑	10 µM	Riu et al. [109]
			*p,p’*-DDE	adipogenesis (−) lipid accumulation (−) (2 µM, 20 µM) basal fatty acid uptake ↑ (2 µM) insulin-stimulated fatty acid uptake (−) (2 µM, 20 µM) lipolysis (−) (2 µM, 20 µM) leptin, resistin, adiponectin release ↑ (2 µM, 20 µM) mRNA level of *adiponectin*, *resistin* ↑ (20 µM)	2 µM, 20 µM	Howell et al. [80]
			oxychlordane	adipogenesis (−) lipid accumulation (−) (2 µM, 20 µM) basal fatty acid uptake ↑ (20 µM) insulin-stimulated fatty acid uptake (−) (2 µM, 20 µM) lipolysis (−) (2 µM, 20 µM)	20 µM	Howell et al. [80]
			dieldrin	adipogenesis (−) (2 µM), ↓ (20 µM) lipid accumulation (−) (2 µM), ↓ (20 µM) basal fatty acid uptake ↑ (2 µM, 20 µM) insulin-stimulated fatty acid uptake (−) (2 µM, 20 µM) lipolysis (−) (2 µM, 20 µM) adiponectin release ↑ (2 µM)	2 µM, 20 µM	Howell et al [80]
		**3T3-F442A cell line**	BPA	basal glucose uptake ↑ (10^−4^ M) insulin-stimulated glucose uptake ↑ (10^−4^ M, 10^−6^ M) GLUT4 protein expression ↑ (10^−4^ M, 10^−6^ M)	10^−4^ M, 10^−6^ M	Sakurai et al. [81]
			*p,p’*-DDT	adipogenesis ↓ (most cells did not differentiate completely) C/EBPα protein level ↓	20 µM	Moreno-Aliaga and Matsumura [111]
		**OP9 cell line**	TBT	preadipocyte differentiation ↑ triglyceride accumulation ↑	100 nM	Kassotis et al. [39]
			TBBPA	preadipocyte differentiation ↑ triglyceride accumulation ↑	10 µM	Kassotis et al. [39]
				adipogenesis ↑ lipid accumulation ↑ (20 µM) PPARγ activity ↑ (10, 20 µM)	20 µM	Andrews et al. [120]
			TCBPA	preadipocyte differentiation ↑ triglyceride accumulation ↑	10 µM	Kassotis et al. [39]
		**ST-13 cell line**	TBBPA	undifferentiated cells: lipid accumulation (−) (0.5 µM, 1 µM) mRNA level of *AACS*, *PLIN1*, *FAS*, *CIDEA*, *LSD-1* ↑ (0.5 µM, 1 µM); *UCP-1*, *UCP-3*, *PRDM16* ↑ (1 µM); *SCOT*, *PPARγ* (−) (0.5 µM, 1 µM) mature adipocytes: lipid accumulation (−) (0.5 µM, 1 µM) mRNA level of *AACS*, *SCOT* (−) (0.5 µM, 1 µM)	0.5 µM, 1 µM	Yamasaki et al. [82]
		**UCP-1** **cell line**	CPF	mRNA level of *UCP1*, *CPT1A*, *CPT1B*, *ACAT3*, *COX16* ↓ mRNA level of *PPARγ*, *PPARGC1A*, *PRDM16* (−) mitochondrial respiration ↓	1 pM	Wang et al. [29]
	**Human**	**Primary preadipocytes**	BPA	adipogenesis ↑ lipid accumulation ↑ (25 µM, 50 µM) mRNA level of *AP2*, *C/EBPα* ↑ (25 µM, 50 µM), *adipsin*, *PPARγ*, *C/EBPβ* ↑ (50 µM) protein level of AP2 ↑ (25 µM) probable adipogenic action via a non-classical estrogen-receptor (ER) pathway rather than through the glucocorticoid-receptor (GR) activation	25 µM, 50 µM	Boucher, Boudreau, and Atlas [75]
				preadipocyte differentiation ↑ expression of genes *ACACA*, *APOA1BP*, *PLIN2*, *FADS1*, *NPC2*, *PPAP2A* ↑ mRNA level of *SREBF1*, *LDLR*, *LPL*, *INSIG1*, *GDF15* ↑ probable mechanism of action via mTOR signaling and TR/RXR activation	50 µM	Boucher et al. [70]
				adipogenesis ↑ lipid accumulation ↑ (10 nM, 1 µM, 80 µM) mRNA level of *11β-HSD1*, *PPAR*γ ↑ (10 nM, 1 µM, 80 µM), *LPL* ↑ (10 nM, 80 µM) probable mechanism of action through GR pathway	10 nM, 1 µM, 80 µM	Wang et al [76]
				adipogenesis ↑ lipid accumulation ↑ (1 nM, 10 nM) mRNA level of *ERα* ↑ (10 nM, 100 nM), *ERRγ* ↑ (10 nM), *LEP* ↑ (10 nM, 100 nM), *ERβ* (−) (1 nM, 10 nM, 100 nM), *GPR30* ↓ (10 nM) the expression of *CD36*, *FABP4* ↑ (1 nM, 10 nM) secretion of IL1B, IL18, CCL20 ↑ (10 nM)	1 nM, 10 nM, 100 nM	Menale et al. [124]
		**PCS-210-010 cells**	BPA	adipogenesis ↑ mRNA level of *PPARγ*, *AP2*, *PPIA* ↑ adiponectin release ↑	0.059 µM	El-Atta et al. [83]
		**SGBS cells**	BPA	lipid accumulation ↓ (10 nM, 100 nM, 1 µM, 10 µM) binding to PPARγ ↑ (50 µM) PPARγ activity (−) (10 nM, 100 nM, 1 µM, 10 µM) protein level of FABP4 ↓ (10 nM, 100 nM, 1 µM), GPD1 ↓ (10 nM, 100 nM, 1 µM), LPL ↓ (100 nM, 1 µM), APOE ↓ (10 nM, 100 nM, 1 µM, 10 µM) protein level of LIPE ↑ (10 nM, 100 nM, 1 µM, 10 µM), PNPLA2 ↑ (10 nM, 100 nM, 10 µM), CD36 ↑ (10 nM, 100 nM, 1 µM, 10 µM), ADIPOQ ↑ (10 nM, 100 nM, 10 µM) release of MCP1 ↑ (1 µM), leptin ↑ (10 nM) proteins related to oxidative stress level of CAT ↓ (100 nM, 1 µM), SOD2 ↓ (10 nM, 100 nM, 1 µM, 10 µM) cellular ROS level ↓ (10 nM, 100 nM, 1 µM, 10 µM) insulin sensitivity, pAKT/AKT ratio ↓ (1 µM)	10 nM, 100 nM, 1 µM, 10 µM, 50 µM	Schaffert et al. [8]
			BPS	lipid accumulation ↓ (10 nM, 100 nM, 1 µM, 10 µM) binding to PPARγ ↑ (50 µM) PPARγ activity (−) (10 nM, 100 nM, 1 µM, 10 µM) protein level of FABP4 ↓ (100 nM, 1 µM), GPD1 ↓ (10 nM, 100 nM, 1 µM, 10 µM), LPL ↓ (100 nM, 1 µM, 10 µM), APOE ↓ (10 nM, 100 nM, 1 µM, 10 µM) protein level of LIPE ↑ (10 nM, 1 µM, 10 µM), PNPLA2 ↑ (10 nM, 100 nM, 1 µM, 10 µM), CD36 ↑ (10 nM, 1 µM, 10 µM), ADIPOQ ↑ (10 nM, 1 µM, 10 µM) release of adiponectin ↓ (1 µM), LEP ↑ (10 nM) proteins related to oxidative stress level of CAT ↓ (10 nM, 100 nM), SOD1 ↓ (10 µM), SOD2 ↓ (10 nM, 100 nM, 1 µM, 10 µM) cellular ROS level ↓ (10 nM, 100 nM, 1 µM, 10 µM) insulin sensitivity, pAKT/AKT ratio ↓ (1 µM)	10 nM, 100 nM, 1 µM, 10 µM, 50 µM	Schaffert et al. [8]
			BPB	lipid accumulation ↓ (10 nM, 100 nM, 1 µM, 10 µM) binding to PPARγ ↑ (50 µM) PPARγ activity (−) (10 nM, 100 nM, 1 µM, 10 µM) protein level of FABP4, GPD1, LPL, APOE, ADIPOQ ↓ (10 nM, 100 nM, 1 µM, 10 µM) protein level of PNPLA2 ↑ (10 nM), CD36 ↑ (10 nM, 10 µM) release of adiponectin ↓, MCP1 ↑, leptin ↓ (1 µM) proteins related to oxidative stress level of CAT ↓ (10 nM, 100 nM, 1 µM, 10 µM), SOD1 ↓ (10 nM, 100 nM, 1 µM, 10 µM), SOD2 ↓ (10 nM, 100 nM, 1 µM) cellular ROS level ↓ (10 nM, 100 nM, 1 µM, 10 µM) insulin sensitivity, pAKT/AKT ratio ↓ (1 µM)	10 nM, 100 nM, 1 µM, 10 µM, 50 µM	Schaffert et al. [8]
			BPF	lipid accumulation ↓ (10 nM, 100 nM, 1 µM, 10 µM) binding to PPARγ ↑ (50 µM) PPARγ activity (−) (10 nM, 100 nM, 1 µM, 10 µM) protein level of FABP4, GPD1, LPL, APOE ↓ (10 nM, 100 nM, 1 µM, 10 µM) protein level of LIPE ↑ (1 µM, 10 µM), PNPLA2 ↑ (10 µM), CD36 ↑ (10 nM, 100 nM, 1 µM, 10 µM), ADIPOQ ↑ (10 nM, 10 µM) release of adiponectin ↓ (1 µM), MCP1 ↑ (1 µM), leptin ↑ (10 nM, 1 µM) proteins related to oxidative stress level of CAT ↓ (10 nM, 100 nM, 1 µM, 10 µM), SOD1 ↓ (10 nM, 100 nM, 10 µM), SOD2 ↓ (100 nM, 1 µM, 10 µM) cellular ROS level ↓ (10 nM, 100 nM, 1 µM, 10 µM)	10 nM, 100 nM, 1 µM, 10 µM 50 µM	Schaffert et al. [8]
			BPAF	lipid accumulation ↓ (10 nM, 100 nM, 1 µM) binding to PPARγ ↑ (50 µM) PPARγ activity (−) (10 nM, 100 nM, 1 µM, 10 µM) protein level of FABP4, GPD1, LPL, APOE ↓ (10 nM, 100 nM, 1 µM) protein level of LIPE ↑ (10 nM, 100 nM), CD36 ↑ (10 nM, 1 µM), ADIPOQ ↑ (1 µM) release of adiponectin ↓ (1 µM), leptin ↑ (10 nM) proteins related to oxidative stress level of CAT ↓ (100 nM), SOD1 ↓ (10 nM, 100 nM, 1 µM), SOD2 ↓ 10 nM, 100 nM, 1 µM) cellular ROS level ↓ (10 nM, 100 nM, 1 µM)	10 nM, 100 nM, 1 µM, 50 µM	Schaffert et al. [8]
			DEHP	triglyceride content ↓ mRNA level of *ADIPOR2*, *GLUT4* (−), *LEPR*, *CD36*, *FABP4*, *LPL*, *LIPE*, *ATGL* ↓ protein level of PPARα, PPARγ, SOD2, GPX1 (−) secretion of adiponectin ↓, LEP ↑ ratio of pAMPK/AMPK, pSTAT3/STAT3 (−), pACC2/ACC2 ↑ phosphorylation of ERK1, ERK2 ↓ lipolysis ↑, level of free glycerol ↑ ROS level ↑	50 µg/mL	Schaedlich et al. [84]
			DINCH	binding to PPARγ ↑ (−) (6.25 µM, 12.5 µM, 25 µM, 50 µM, 100 µM, 200 µM, 400 µM) PPARγ activation (−) undifferentiated cells: lipid accumulation (−) (10 nM, 1 µM, 10 µM, 25 µM, 50 µM, 100 µM) secretion of adipsin ↑ (10 nM), MCP-1 ↓ (10 µM) mature adipocytes: lipid accumulation ↓ (10 µM, 25 µM, 50 µM, 100 µM) secretion of LEP ↑ (10 nM, 10 µM), adipsin ↑ (10 nM, 10 µM), MCP-1 ↑ (10 nM, 10 µM), adiponectin ↓ (10 nM, 10 µM) protein level of GPX1 ↑ (10 µM), GPX4 ↑ (10 µM), GSTO1 ↑ (10 nM, 10 µM), LAP3 ↑ (10 µM)	10 nM, 10 µM, 25 µM, 50 µM, 100 µM	Schaffert et al. [62]
			DINP	binding to PPARγ ↑ (−) (6.25 µM, 12.5 µM, 25 µM, 50 µM, 100 µM, 200 µM, 400 µM, PPARγ activation (−) undifferentiated cells: lipid accumulation (−) (10 nM, 1 µM, 10 µM, 25 µM, 50 µM, 100 µM), secretion of adipsin ↑ (10 nM) mature adipocytes: lipid accumulation (−) (10 nM, 1 µM, 10 µM, 25 µM, 50 µM, 100 µM), secretion of LEP ↑ (10 nM, 10 µM), adipsin ↑ (10 µM), MCP-1 ↑ (10 µM), adiponectin ↓ (10 nM, 10 µM) protein level of GPX1 ↑ (10 µM), GPX4 ↑ (10 nM, 10 µM), GPX8 ↑ (10 nM), GSR ↑ (10 nM), GSTO1 ↑ (10 nM, 10 µM), LAP3 ↑ (10 µM)	10 nM, 10 µM	Schaffert et al. [62]
			DPHP	binding to PPARγ ↑ (−) (6.25 µM, 12.5 µM, 25 µM, 50 µM, 100 µM, 200 µM, 400 µM), PPARγ activation (−) undifferentiated cells: lipid accumulation (−) (10 nM, 1 µM, 10 µM, 25 µM, 50 µM, 100 µM), secretion of MCP-1 ↓ (10 nM, 10 µM) mature adipocytes: lipid accumulation ↓ (10 µM, 25 µM, 50 µM, 100 µM) secretion of LEP ↑ (10 µM), adipsin ↑ (10 µM), MCP-1 ↑ (10 µM), adiponectin ↓ (10 nM, 10 µM) protein level of GPX1 ↑ (10 nM, 10 µM), GPX4 ↑ (10 µM), GPX8 ↑ (10 µM), GSR ↑ (10 nM), GSTO1 ↑ (10 nM, 10 µM), LAP3 ↑ (10 µM)	10 nM, 10 µM, 25 µM, 50 µM, 100 µM	Schaffert et al. [62]
			MINCH	binding to PPARγ ↑ (25 µM, 50 µM, 100 µM, 200 µM, 400 µM) PPARγ activation (−) undifferentiated cells: preadipocyte differentiation ↑ lipid accumulation ↑ (10 µM, 25 µM, 50 µM) secretion of LEP ↑ (10 µM), adipsin ↑ (10 nM), MCP-1 ↓ (10 nM, 10 µM) protein level of FABP4, FASN, FABP5, GPD1, PLIN1 ↑ (10 µM) mature adipocytes: lipid accumulation ↓ (10 µM, 25 µM, 50 µM) secretion of LEP ↑ (10 nM, 10 µM), adipsin ↑ (10 µM), MCP-1 ↑ (10 nM, 10 µM), adiponectin ↓ (10 nM, 10 µM) protein level of GPX1 ↑ (10 µM), GPX4 ↑ (10 µM), GPX8 ↑ (10 nM, 10 µM), GSTO1 ↑ (10 nM, 10 µM), LAP3 ↑ (10 µM)	10 nM, 10 µM, 25 µM, 50 µM, 100 µM, 200 µM, 400 µM	Schaffert et al. [62]
			MHINP	binding to PPARγ ↑ (100 µM, 200 µM, 400 µM) PPARγ activation ↑ (1 µM) undifferentiated cells: preadipocyte differentiation ↑ lipid accumulation ↑ (10 µM, 25 µM, 50 µM, 100 µM) secretion of LEP ↑ (10 µM), adipsin ↑ (10 nM, 10 µM), MCP-1 ↓ (10 nM) protein level of FABP4, FASN, FABP5, GPD1, PLIN1 ↑ (10 µM) mature adipocytes: lipid accumulation ↑ (1 µM) secretion of LEP ↑ (10 nM, 10 µM), adipsin ↑ (10 µM), MCP-1 ↑ (10 nM, 10 µM), adiponectin ↓ (10 nM, 10 µM) protein level of GPX1 ↑ (10 nM, 10 µM), GPX4 ↑ (10 nM, 10 µM), GPX8 ↑ (10 nM), GSR ↑ (10 nM), GSTO1 ↑ (10 nM, 10 µM), LAP3 ↑ (10 µM)	10 nM, 10 µM, 25 µM, 50 µM, 100 µM, 200 µM, 400 µM	Schaffert et al. [62]
			OH-MPHP	binding to PPARγ ↑ (200 µM, 400 µM) PPARγ activation ↑ (10 µM) undifferentiated cells: preadipocyte differentiation ↑ lipid accumulation ↑ (10 µM, 25 µM, 50 µM) secretion of LEP ↑ (10 µM) protein level of FABP4, FASN, FABP5, GPD1 ↑ (10 µM) mature adipocytes: lipid accumulation ↓ (10 nM, 10 µM, 25 µM, 50 µM, 100 µM) secretion of LEP ↑ (10 nM, 10 µM), adipsin ↑ (10 µM), MCP-1 ↑ (10 nM, 10 µM), adiponectin ↓ (10 nM, 10 µM) protein level of GPX1 ↑ (10 µM), GPX4 ↑ (10 µM), GPX8 ↑ (10 µM), GSR ↑ (10 nM, 10 µM), GSTO1 ↑ (10 nM, 10 µM), LAP3 ↑ (10 µM)	10 nM, 10 µM, 25 µM, 50 µM, 100 µM, 200 µM, 400 µM	Schaffert et al. [62]
		**SW 872** **cell line**	MEHP	differentiating effect ↑ lipid accumulation (−) mRNA level of *TSPO*, *PKCε*, *PPARα*, *ACACA*, *ACLY*, *GLUT1*, *GLUT4*, *S100B* ↑, *PPARγ* ↓, *PPARβ/δ* (−) protein level of TSPO ↓	10 µM	Campioli et al. [129]

Legend: ↑ increase; ↓ decrease; (−) no observed effects; * concentration (s) at which obesogenic effects were observed.

**Table 2:** The obesogenic effect of selected EDCs confirmed on mesenchymal stem cell models.

Cell Type	Organism	In vitro Model	EDCs	Mechanism of Action	Concentration *	References
**Adipose-derived mesenchymal stem cells (ADSCs)**	**Animal**	**C3H10T1/2** **cell line**	BPA	adipogenic differentiation (− (1 nM, 3 nM) lipid accumulation (−) (1 nM, 3 nM) mRNA level of *PPARγ*, *C/EBPα* and *FABP4* (−) (1 nM, 3 nM)	1 nM, 3 nM	De Filippis et al. [96]
				whole period of adipogenic differentiation: amount of adipocytes ↓, triglyceride content ↓, mRNA level of *FABP4*, *PPARγ*, *LPL*, *adiponectin* ↓ (10 µM); amount of adipocytes (−), triglyceride content (−) (10 nM) selective treatment during undifferentiated growth: amount of adipocytes ↓, triglyceride content ↓ (10 µM); hormonal induction: amount of adipocytes (−), triglyceride content (−) (10 µM); terminal differentiation: amount of adipocytes (−), triglyceride content (−) (10 µM)	10 µM	Biemann et al. [139]
			DEHP	whole period of adipogenic differentiation: amount of adipocytes ↑, triglyceride content ↑ (100 µM); amount of adipocytes (−), triglyceride content (−) (100 nM) selective treatment during undifferentiated growth: amount of adipocytes (−), triglyceride content (−) (100 µM); hormonal induction: amount of adipocytes ↑, triglyceride content ↑ (100 µM); terminal differentiation: amount of adipocytes (−), triglyceride content (−) (100 µM)	100 µM	Biemann et al. [139]
			BBP	adipogenesis ↑ lipid accumulation ↑ mRNA level of *AP2*, *PPARγ* ↑, *SIRT1*, *SIRT7*, *PGC1α*, *NRF1*, *NRF2*, *TFAM* ↓; *SIRT2*, *SIRT3*, *SIRT4*, *SIRT5*, *SIRT6* (−) protein level of FOXO1 ↑, SIRT1, SIRT3 ↓, SIRT7, β-catenin (−) acetylation of FOXO1, β-catenin ↑	50 µM	Zhang and Choudhury [138]
				expression of miR-103/107, miR-let7 (a, b, c, d, f, g) ↑, lncRNA H19 ↓, miR-let7e (−) at day 2 of differentiation mRNA level of *IRS-1* ↓, *IR*, *IRS-2* (−) at day 2, 4, 6, 8 of differentiation protein expression of phospho-Akt ↓ at day 4 of differentiation	50 µM	Zhang and Choudhury [137]
			TBT	whole period of adipogenic differentiation: amount of adipocytes ↑, triglyceride content ↑ (100 nM); amount of adipocytes (−), triglyceride content (−) (1 nM) selective treatment during undifferentiated growth: amount of adipocytes ↑, triglyceride content ↑ (100 nM); hormonal induction: amount of adipocytes ↑, triglyceride content ↑ (100 nM); terminal differentiation: amount of adipocytes ↑, triglyceride content ↑ (100 nM)	100 nM	Biemann et al. [139]
			butylparaben	adipogenic differentiation ↑ lipid accumulation ↑ PPARγ activity ↑ GR activity (−) mRNA level of *PPARγ*, *C/EBPα*, *FABP4* ↑, *RUNX2* ↓	100 µM	Hu et al. [130]
			mixture of BPA, DEHP, TBT	BPA (10 nM), DEHP (100 nM) and TBT (1 nM) whole period of adipogenic differentiation, undifferentiated growth, hormonal induction and terminal differentiation: amount of adipocytes (−), triglyceride content (−), mRNA level of *FABP4*, *adiponectin*, *LPL*, *PPARγ2* (−) BPA (10 µM), DEHP (100 µM) and TBT (100 nM) whole period of adipogenic differentiation: amount of adipocytes ↑, triglyceride content (−), mRNA level of *FABP4*, *adiponectin* ↑, *LPL*, *PPARγ2* (−); undifferentiated growth: amount of adipocytes ↑, triglyceride content (−), mRNA level of *FABP4* ↑, *adiponectin*, *LPL*, *PPARγ2* (−); hormonal induction: amount of adipocytes ↑, triglyceride content (−), mRNA level of *FABP4*, *adiponectin*, *LPL*, *PPARγ2* ↑; terminal differentiation: amount of adipocytes ↑, triglyceride content (−); mRNA level of *FABP4* ↑, *adiponectin*, *Lpl*, *PPARγ2* (−)	1 nM, 10 nM, 100 nM, 10 µM, 100 µM	Biemann, Fisher and Navarrete Santos [140]
		**Primary mouse ADSCs**	TBT	adipogenesis ↑ lipid accumulation ↑ mRNA level of *FABP4*, *PPARΓ2*, *LEP* ↑, *PREF-1* ↓, *ADIPOQ* (−)	50 nM	Kirchner et al. [78]
		**Primary porcine ADSCs**	BPS	viability of cells ↓ lipid accumulation ↓	1 µM	Berni et al. [143]
			GLY	viability of cells ↓ adipogenic differentiation ↓	4 µg/mL	Gigante et al. [142]
	**Human**	**Primary hADSCs**	BPA	adipogenic differentiation (−) (1 nM, 3 nM) lipid accumulation (−) (1 nM, 3 nM) mRNA level of *PPARγ*, *C/EBPα* and *FABP4* (−) (1 nM, 3 nM)	1 nM, 3 nM	De Filippis et al. [96]
				mRNA level of *PPARγ*, *GLUT4* (−) insulin-stimulated glucose utilization ↓ insulin-activated insulin receptor (IR) tyrosine phosphorylation ↓, ERK1/2 phosphorylation ↓, PKB/Akt phosphorylation ↓ release of IL6, IFN-γ ↑ JAK/STAT, JNK, NFkB pathways activity ↑	1 nM	Valentino et al. [147]
				adipogenesis ↑ lipid accumulation ↑ mRNA level of *ERα ERβ*, *IGF1*, *PPARγ*, *LPL*, *C/EBPα*, *DLK* ↑, *AP2*, *SREBP1c*, *C/EBPβ* (−) protein level of LPL ↑ probable mechanism of action through ER-dependent pathway	1 µM	Ohlstein et al. [148]
				adipogenesis ↑ (0.1 µM), ↓ (1 µM) lipid production ↑ (0.1 µM), ↓ (1 µM)	0.1 µM, 1 µM	Cohen et al. [55]
				cell viability ↓ (1 µM, 10 µM) apoptosis ↑ (1 µM) caspase-6 activity (1 µM)	1 µM, 10 µM	Harnett et al. [56]
			BPF	adipogenesis ↑ lipid accumulation ↑ (10 µM, 25 µM) mRNA level of *PPARγ*, *LPL*, *FABP4* ↑ (10 µM, 25 µM); *C/EBPα* ↑ (1 µM, 10 µM, 25 µM) protein level of PPARγ, C/EBPα, LPL ↑ (10 µM, 25 µM) probable mechanism of action via an ER-dependent pathway	1 µM, 10 µM, 25 µM	Reina-Pérez et al. [79]
			BPS	adipogenesis ↑ lipid accumulation ↑ (1 µM, 10 µM, 25 µM) mRNA level of *PPARγ*, *C/EBPα*, *LPL* ↑ (1 µM, 10 µM, 25 µM); *FABP4* ↑ (10 µM, 25 µM) protein level of PPARγ ↑ (10 µM, 25 µM); C/EBPα, LPL ↓ (10 µM, 25 µM)	1 µM, 10 µM, 25 µM	Reina-Pérez et al. [79]
			BPAF	adipogenesis ↑ (0.1 nM), (−) (1 nM), ↓ (10 nM) lipid production ↑ (0.1 nM), (−) (1 nM), ↓ (10 nM)	0.1 nM, 10 nM	Cohen et al. [55]
				cell viability ↓ (0.0003 µM, 0.003 µM, 0.03 µM, 0.3 µM) apoptosis ↑ (0.003 µM) caspase-6 activity (0.003 µM)	0.0003 µM, 0.003 µM, 0.03 µM, 0.3 µM	Harnett et al. [56]
			TMBPF	adipogenesis ↓ lipid production ↓	0.01 µM, 0.1 µM	Cohen et al. [55]
				cell viability ↓ (0.01 µM, 0.1 µM, 1 µM, 10 µM, 50 µM) apoptosis ↑ (1 µM) caspase-6 activity (1 µM)	0.01 µM, 0.1 µM, 1 µM, 10 µM, 50 µM	Harnett et al. [56]
			TBT	adipogenesis ↑ lipid accumulation ↑ mRNA level of *FABP4*, *PPARγ2*, *LEP* ↑, *PREF-1* ↓, *ADIPOQ* (−)	50 nM	Kirchner et al. [78]
			p,p’-DDE	maintenance of cells in an undifferentiated state ↑ mRNA level of *OCT4*, *PPARγ*, *SREBP1*, *FASN*, *INSR*, *AKT2*, *UCP-3* ↑ (0.1 µM, 1 µM, 10 µM); *SOX2* ↑ (1 µM, 10 µM); *PPARγC1B* ↑ (0.1 µM, 1 µM); *NANOG* ↓ (0.1 µM, 1 µM, 10 µM)	0.1 µM, 1 µM, 10 µM	Pesta et al. [77]

Legend: ↑ increase; ↓ decrease; (−) no observed effects; * concentration (s) at which biological effects were observed.

**Table 3:** The obesogenic effects of selected EDCs confirmed in hepatic cellular models.

Cell Type	Organism	In vitro Model	EDC	Mechanism of Action	Concentration *	References
**Hepatocytes**	**Animal**	Primary mouse hepatocytes	BHPF	cell viability ↓ LDH activity ↑	1 µM, 10 µM	Yang et al. [165]
		**Primary gilthead seabream hepatocytes**	DIDP	binding to PPARγ, PPARα, RXRα ↑ mRNA level of *PPARA*, *PPARΒ*, *PPARγ*, *CPT1A*, *FADS2*, *SCD1A*, *SCD1B*, *LPL*, *HL*, *FABP*, *APOA-I*, *SREBP1* ↑ (0.1 µM, 1 µM), (−) (10 µM), *CPT1B* ↑ (1 µM), (−) (10 µM) probable mechanism of action via PPAR:RXR signaling	0.1 µM, 1 µM	Cocci et al. [169]
		**Primary Atlantic salmon hepatocytes**	p,p’-DDE	viability of cells ↓ (100 μM), (−) (0.1 μM, 1 μM, 10 μM) global DNA methylation (−) (0.1 μM, 1 μM, 10 μM, 100 μM) mRNA level of stress-responsive genes such as estrogenic markers *ESR1* ↑ (10 μM), (−) (0.1 μM, 1 μM, 100 μM); *ESR2* (−) (0.1 μM, 1 μM, 10 μM, 100 μM); *VTG1* ↑ (1 μM, 10 μM), (−) (0.1 μM, 100 μM); markers of detoxification *CYP1A1*, *CYP3A* (−) (0.1 μM, 1 μM, 10 μM, 100 μM); genes whose protein products are associated with cell death *HSPA8*, *FOS* ↑ (100 μM), (−) (0.1 μM, 1 μM, 10 μM); *CASP3B*, *PTGS2* ↓ (100 μM), (−) (0.1 μM, 1 μM, 10 μM); *CDKN1B*, *INSIG1* (−) (0.1 μM, 1 μM, 10 μM, 100 μM) mRNA level of DNA methylation-relevant genes *DNMT3AA*, *CBS*, *N6AMT2*, *MAT1A2* ↓ (100 μM), (−) (0.1 μM, 1 μM, 10 μM); *DNMT1* (−) (0.1 μM, 1 μM, 10 μM, 100 μM) bile acid metabolism (level of glycocholate, glycochenodeoxycholate, taurocholate, taurochenodeoxycholate, deoxycholate, glycodeoxycholate, taurolithocholate ↑ (100 μM)) glucose metabolism (level of glycolytic intermediates such as 3-phosphoglycerate, phosphoenolpyruvate, glucose-6-phosphate, glucose ↓, pyruvate ↑, pentose sugars such as arabonate/xylonite, arabitol/xylitol, sedoheptulose ↓, pentose phosphate pathway intermediate such as 5-phosphogluconate ↓, glycogen hydrolysis products such as maltotriose, maltotetraose, maltose ↓ (100 μM)) amino acids metabolism (level of glutamine, N-acetylglutamine, glutamate, N-acetylglutamate ↓, gamma-aminobutyrate ↑, (100 μM)) lipid metabolism (level of monoacylglycerols such as 1-mirystoylglycerol ↑, diacylglycerols such as 1-palmitoyl-2-arachidononyl-GPE, phosphatidylethanolamine species such as oleoyl-oleoyl-glycerol ↓ (100 μM))	1 μM, 10 μM, 100 μM	Olsvik and Søfteland [170]
		**BRL-3A** **cell line**	MEHP	lipid accumulation ↑ (100 µM, 200 µM) mRNA level of *FAS*, *PDK4*, *aP2* ↑ (10 µM, 50 µM, 100 µM, 200 µM); *AOX*, *PPARγ* ↑ (50 µM, 100 µM, 200 µM); *JAK2*, *STAT5A* ↓ (50 µM, 100 µM, 200 µM), *STAT5B* ↓ (10 µM, 50 µM, 100 µM, 200 µM) protein level of AOX, PDK4, FAS ↑ (100 µM, 200 µM); aP2 ↑ (50 µM, 200 µM; PPARγ ↑ (200 µM); JAK2, STAT5A, STAT5B ↓ (10 µM, 50 µM, 100 µM, 200 µM); JAK2/STAT5 signalling ↓ level of indicators of oxidative stress: SOD ↓, MDA ↑ (10 µM, 50 µM, 100 µM, 200 µM) level of indicators of damage status of liver cells: ALT ↑ (50 µM, 100 µM, 200 µM), AST ↑ (10 µM, 50 µM, 100 µM, 200 µM)	10 µM, 50 µM, 100 µM, 200 µM	Zhang et al. [173]
		**FaO cell line**	BPA	intracellular lipid content ↑ (30 ng/mL, 300 ng/mL) mRNA level of *PPARα*, *PPARβ*, *PPARδ*, *PPARγ*, *AOX*, *CPT1*, *APOB* ↓, *FAS*, *GPAT* (−) (300 ng/mL)	30 ng/mL and 300 ng/mL corresponding to 0.1 µM and 1 µM	Grasselli et al. [171]
		**AML12** **cell line**	PCB-153	lipid accumulation ↑ mRNA level of p65 subunit of *NFkB, IL1α*, *IL6* ↑, *HNF1B*, *GPX1* ↓ nuclear protein expression of p65 subunit of NFkB ↑, cytoplasmic protein expression of p65 subunit of NFkB ↓ protein level of HNF1b, GPX1 ↓ ROS level ↑ ratio of glutathione (GSH)/ /oxidized GSH (GSSG) ↓ ratio of NADP+/NADPH ↑ insulin-stimulated glucose uptake ↓	0.5 µM, 1 µM	Wu et al. [175]
			TCEP	lipid accumulation ↑ (10 µM) disturbance of mitochondrial structure ↑ (1 µM, 10 µM) mitoATP rate/glycoATP rate ↓ (1 µM, 10 µM)	1 µM, 10 µM	Le et al [176]
			TCPP	lipid accumulation ↑ (10 µM) disturbance of mitochondrial structure ↑ (1 µM, 10 µM) mitochondrial membrane potential (MMP) ↓ (10 µM) mitoATP rate/glycoATP rate ↓ (10 µM)	1 µM, 10 µM	Le et al. [176]
			TDCPP	lipid accumulation ↑ (1 µM, 10 µM) disturbance of mitochondrial structure ↑ (1 µM, 10 µM) mitochondrial ROS production ↑ (1 µM, 10 µM) MMP ↓ (10 µM) mitochondrial basal respiration ↓ (10 µM) proton leak ↓ (10 µM) mitoATP production rate ↓ (10 µM) mitoATP rate/glycoATP rate ↓ (10 µM)	1 µM, 10 µM	Le et al. [176]
			TPhP	lipid accumulation ↑ (1 µM, 10 µM) disturbance of mitochondrial structure ↑ (1 µM, 10 µM) mitochondrial ROS production ↑ (1 µM, 10 µM) MMP ↓ (1 µM, 10 µM) mitochondrial basal respiration ↓ (10 µM) proton leak ↓ (10 µM) spare respiratory capacity (SRC) ↑ (10 µM) mitoATP production rate ↓ (10 µM) mitoATP rate/glycoATP rate ↓ (10 µM)	1 µM, 10 µM	Le et al. [176]
			TCP	lipid accumulation ↑ (0.1 µM, 1 µM, 10 µM) disturbance of mitochondrial structure ↑ (1 µM, 10 µM) mitochondrial ROS production ↑ (1 µM, 10 µM) MMP ↓ (1 µM, 10 µM) mitochondrial basal respiration ↓ (10 µM) proton leak ↓ (10 µM) SRC ↑ (10 µM) mitoATP production rate ↓ (10 µM) mitoATP rate/glycoATP rate ↓ (10 µM)	0.1 µM, 1 µM, 10 µM	Le et al. [176]
		**Hepa1-6** **cell line**	BPA	mRNA level of DNA methyltransferases: *DNMT1*, *DNMT3A* ↓ (0.001 µM, 0.01 µM); *DNMT3B* ↓ (0.001 µM) mRNA level of *SREBF1*, *SREBF2*, *FASN*, *HMGCR* ↑ (0.001 µM, 0.01 µM)	0.001 µM, 0.01 µM	Ke et al. [178]
		**FL83B** **cell line**	DEHP	cell viability ↓ (250 µM, 500 µM, 1000 µM) LDH release ↑ (125 µM, 250 µM, 500 µM, 1000 µM), ALT release ↑ (500 µM, 1000 µM) cell populations of sub-G1 and S phase ↑ (250 µM, 500 µM, 1000 µM)	125 µM, 250 µM, 500 µM, 1000 µM	Lo et al. [185]
		**RTL-W1** **cell line**	BPA	lipid accumulation ↑ alteration of membrane lipids (phosphatidylcholines (PCs), plasmalogen PCs) mRNA level of *ABCA1*, *CD36*, *FATP1*, *FAS*, *LPL*, *PPARα*, *PPARβ* ↑	10 µM	Dimastrogiovanni et al. [186]
			TBT	alteration of membrane lipids (phosphatidylcholines (PCs), plasmalogen PCs) mRNA level of *ABCA1*, *CD36*, *FAS*, *LPL* ↑	100 nM	Dimastrogiovanni et al. [186]
			TPT	mRNA level of *ABCA1*, *FATP1*, *FAS* ↑	100 nM	Dimastrogiovanni et al. [186]
			DEHP	lipid accumulation ↑ alteration of membrane lipids (phosphatidylcholines (PCs), plasmalogen PCs) mRNA level of *CD36*, *FAS*, *LPL* ↓	5 µM	Dimastrogiovanni et al. [186]
			4-NP	alteration of membrane lipids (phosphatidylcholines (PCs), plasmalogen PCs) mRNA level of *ABCA1* ↑, *CD36*, *FAS*, *LPL*, *PPARβ* ↓	20 µM	Dimastrogiovanni et al. [186]
		**PLHC-1** **cell line**	DBP	triacylglyceride accumulation ↑ (20 µM), (−) (5 µM) ROS generation ↑ (5 µM, 20 µM, 50 µM, 100 µM)	5 µM, 20 µM, 50 µM, 100 µM	Pérez-Albaladejo et al. [191]
			DEHP	triacylglyceride accumulation ↑ (5 µM, 10 µM) ROS generation ↑ (100 µM)	5 µM, 10 µM, 100 µM	Pérez-Albaladejo et al. [191]
			BADGE·2HCl	triacylglyceride accumulation ↓ (1 µM, 5 µM) ROS generation ↑ (100 µM)	1 µM, 5 µM, 100 µM	Pérez-Albaladejo et al. [191]
			TBT	intracellular accumulation of triglycerides and diglycerides ↑	100 nM, 200 nM	Marqueño et al. [188]
		**ZFL cell line**	BPA	lipid accumulation: dihydroceramides ↑ (50 µM), ether-triacylglycerides (ether-TGs), saturated and lower unsaturated TGs ↑ (5 µM, 50 µM) mRNA level of *SCD*, *ELOVL6* ↑, *LXR*, *FASN*, *GPAT3*, *DGAT1A* (−) (20 µM) ROS production ↑ (20 µM, 50 µM, 70 µM, 100 µM, 150 µM, 200 µM)	5 µM, 20 µM, 50 µM, 70 µM, 100 µM, 150 µM, 200 µM	Marqueño et al. [190]
			BPF	lipid accumulation: dihydroceramides, ether-triacylglycerides (ether-TGs) ↑ (50 µM), TGs containing polyunsaturated fatty acids (PUFAs) ↓ (50 µM) mRNA level of *SCD*, *ELOVl6*, *ABCA1B*, *CYP3A65* ↑, *PPARα* ↓, *LXR*, *FASN*, *GPAT3*, *DGAT1a* (−) (20 µM) ROS production ↑ (5 µM, 20 µM, 50 µM, 100 µM, 150 µM, 200 µM, 500 µM)	5 µM, 20 µM, 50 µM, 100 µM, 150 µM, 200 µM, 500 µM	Marqueño et al. [190]
			BADGE·2HCl	lipid accumulation: dihydroceramides, ether-TGs) ↑ (10 µM), saturated and lower unsaturated TGs ↑ (5 µM) mRNA level of *ABCA1b*, *PPARα* ↓, *LXR*, *FASN*, *GPAT3*, *DGAT1A* (−) (5 µM) ROS production (−) (20 µM, 50 µM, 60 µM, 70 µM, 80 µM, 100 µM)	5 µM, 10 µM	Marqueño et al. [190]
	**Human**	**Primary hepatocytes**	TCDD	fatty acids accumulation ↑	10 nM	Forgacs et al. [90]
		**HepG2** **cell line**	4-HP	lipid deposition in OA (oleic acid)-treated cells ↑ *de novo* lipogenesis ↓ fatty acid oxidation ↓ mRNA level of *CD36* ↑ mRNA level of *PPARα*, *SREBP1c*, *CPT1A*, *ACC* ↓, *PPARγ* (−)	20 µM	Sun et al. [93]
			1,3-DCP	cell viability ↓ (250 µg/mL, 500 µg/mL), (−) (0.1 µg/mL, 0.5 µg/mL, 1 µg/mL, 5 µg/mL, 25 µg/mL, 50 µg/mL) intracellular TG and TC (total cholesterol) content ↑ (0.5 µg/mL, 1 µg/mL, 2 µg/mL) level of cAMP, AMP, ADP ↓, ATP (−) (0.5 µg/mL, 1 µg/mL, 2 µg/mL) intracellular calcium level (−) (0.5 µg/mL, 1 µg/mL, 2 µg/mL) mRNA level of *LDLR*, *SREBP2*, *HMGCR* ↑ (0.5 µg/mL, 1 µg/mL, 2 µg/mL) protein level of SREBP1c, SCD1, FAS, CD36, HMGCR, GPAT ↑, CREB, LKB1, HSL, p-AMPK, p-PKA, pACC, PGC1α, PPARα, SIRT1, CPT1, HNF4α ↓ (0.5 µg/mL, 1 µg/mL, 2 µg/mL), GPR41, GPR43 ↓, GPR109B (−) (2 µg/mL), Caldumolin 1, Calpain 1, Calpain 2, CaMKII, p-CaMKII (−) (0.5 µg/mL, 1 µg/mL, 2 µg/mL) Gi/o expression ↑ (2 µg/mL) probable mechanism of action through cAMP/PKA and AMPK signaling pathways via Gi/o-coupled receptor	0.5 µg/mL, 1 µg/mL, 2 µg/mL, 250 µg/mL, 500 µg/mL	Lu et al. [199]
			TCS	level of diacylglycerol (DG) ↑ (1 µM, 10 µM), phosphatidylcholine (PC), sphingomyelin (SM), phosphatidylethanolamine (PE) ↑ (10 µM), ceramide (Cer) ↓ (1 µM, 10 µM), triglyceride (TG), polyphosphoinositide (PI), lysophosphatidylethanolamine (LPE), phosphatidylglycerol (PG), lysophosphatidylcholine (LPC) ↓ (10 µM) ROS generation ↑ (10 µM), (−) (1 µM) MDA content (−) (1 µM, 10 µM) SOD activity ↑ (10 µM), (−) (1 µM) CAT activity ↑ (10 µM), (−) (1 µM) level of GSH ↑ (10 µM), (−) (1 µM), taurine (−) (1 µM, 10 µM)	1 µM, 10 µM	Zhang et al. [213]
			DINCH	cell viability (−) (1 µg/mL, 5 µg/mL, 10 µg/mL, 100 µg/mL, 250 µg/mL, 500 µg/mL) oxidative DNA damage at 3 h exposure ↑ (1 µg/mL, 10 µg/mL, 100 µg/mL, 250 µg/mL, 500 µg/mL), at 24 h exposure ↑ (100 µg/mL)	1 µg/mL, 10 µg/mL, 100 µg/mL, 250 µg/mL, 500 µg/mL	Vasconcelosa, Silva and Louroa [57]
		**HepaRG** **cell line**	BPA	triglycerides and neutral lipids accumulation ↑ (2 nM) mRNA level of lipid-responsive genes such as *APOA4* ↑, *PLIN2* (*ADFP* or *ADRP*), *TIP47* (−) (0.2 nM, 2 nM, 20 nM, 200 nM, 2000 nM) mRNA level of genes involved in lipid and carbohydrate homeostasis *FASN*, *ACLY*, *ACACA*, *HMGCR*, *PPARγ*, *PNPLA3*, *THRSP* (*SPOT14*), *PDK4*, *APOB*, *GLUT2* (*SLC2A2*), *MTTP*, *CPT1A* (−) (0.2 nM, 2 nM, 20 nM, 200 nM, 2000 nM) mRNA level of genes involved in oxidative stress *NFKB1*, *HMOX1*, *GSTA1/2*, *GSTA3*, *NQO1*, *TRIB3*, *HSPA1A* (*HSP70-1A*) (−) (0.2 nM, 2 nM, 20 nM, 200 nM, 2000 nM) mRNA level of ERRγ target genes *SDHD*, *PCK1*, *ESRRA* (−) (0.2 nM, 2 nM, 20 nM, 200 nM, 2000 nM) mRNA level of PXR target genes *CD36*, *CYP2C9*, *CYP3A4* (−) (0.2 nM, 2 nM, 20 nM, 200 nM, 2000 nM) mRNA level of genes involved in BPA biotransformation *CYP2C19*, *CYP2C18*, *SULT1A1*, *SULT1A3/4*, *UGT2B15*, *STS*, *GUSB* (−) (0.2 nM, 2 nM, 20 nM, 200 nM, 2000 nM)	2 nM	Bucher et al. [164]
			TBT	lipid accumulation ↑ (5 nM, 10 nM, 50 nM) mRNA level of *SREBF1*, *FASN* ↑ (50 nM) protein level of RXRα ↓ (50 nM)	5 nM, 10 nM, 50 nM	Stossi et al. [202]
		**Huh-7 cell line**	BPA	cell viability ↓ (200 µM, 400 µM), (−) (10 µM, 100 µM) lipid accumulation ↑ (10 µM, 50 µM, 100 µM, 200 µM), (−) (1 µM) fatty acid uptake ↑ (10 µM, 50 µM, 100 µM), (−) (1 µM) mRNA level of *CD36* ↑ (50 µM, 100 µM), *SR-A1*, *SR-B1* (−) (10 µM, 50 µM, 100 µM) protein level of CD36 ↑ (50 µM, 100 µM), SR-A1, SR-B1 (−) (10 µM, 50 µM, 100 µM) intracellular ROS generation ↑ (10 µM, 50 µM, 100 µM, 200 µM), (−) (1 µM)	10 µM, 50 µM, 100 µM, 200 µM, 400 µM	Lee et al. [206]
		**HHL-5** **cell line**	BPA	lipid accumulation ↑ (10 nM, 100 nM, 1000 nM, 22.5 µM, 45 µM) mRNA level of *SREBP1c*, *ACACA*, *FASN* ↑ (22.5 µM), CNR1 ↑ (45 µM) level of AEA ↑, PEA, OEA ↓ (45 µM) CB1 activity ↑ (45 µM) FAAH activity ↓ (45 µM, 90 µM) probable mechanism of action by endocannabinoid action at CB1 receptors	10 nM, 100 nM, 1000 nM, 22.5 µM, 45 µM, 90 µM	Martella et al. [210]
		**L02 cell line**	TCS	level of TG, PG, LPC, LPE ↑ (1 µM, 2.5 µM), DG, PE, PC, SM, Cer ↑ (2.5 µM), PI ↓ (1 µM, 2.5 µM) ROS generation ↑ (1 µM, 2.5 µM) MDA content ↑ (1 µM, 2.5 µM) SOD activity ↑ (2.5 µM), (−) (1 µM) CAT activity ↑ (1 µM, 2.5 µM) level of GSH ↓ (2.5 µM), (−) (1 µM), taurine ↓ (1 µM, 2.5 µM)	1 µM, 2.5 µM	Zhang et al. [213]

Legend: ↑ increase; ↓ decrease; (−) no observed effects; * concentration (s) at which biological effects were observed.

**Table 4:** The obesogenic effect of selected EDCs confirmed on pancreatic cellular models.

Cell Type	Organism	In Vitro Model	EDCs	Mechanism of Action	Concentration *	References
**Pancreatic cells/islets**	**Animal**	**Rat pancreatic islets**	BPA	acute exposure (60 min) insulin secretion with glucose (8.3 mM or 16.7 mM) stimulation (−) (0.1 µg/L, 1 µg/L, 10 µg/L, 100 µg/L, 1000 µg/L) long-term exposure (24 h) insulin secretion with glucose (16.7 mM) stimulation ↑ (10 µg/L, 100 µg/L) co-incubation of BPA (10 µg/L) with actinomycin-D (Act-D) significantly suppressed insulin secretion probable mechanism of action via cytosolic/nuclear estrogen receptors	10 µg/L, 100 µg/L	Adachi et al. [216]
			NP	acute exposure (60 min) insulin secretion with glucose (8.3 mM or 16.7 mM) stimulation (−) (0.1 µg/L, 1 µg/L, 10 µg/L, 100 µg/L, 1000 µg/L) long-term exposure (24 h) insulin secretion with glucose (16.7 mM) stimulation ↑ (0.1 µg/L, 1 µg/L, 10 µg/L, 100 µg/L) co-incubation of NP (10 µg/L) with Act-D significantly suppressed insulin secretion probable mechanism of action via cytosolic/nuclear estrogen receptors	0.1 µg/L, 1 µg/L, 10 µg/L, 100 µg/L	Adachi et al. [216]
			TBT	viability of cells ↓ (0.01 µM, 0.1 µM, 1 µM, 10 µM, 100 µM) ↑ (10 µM) insulin secretion at both basal (2.8 mM) and stimulatory (16.7 mM) concentrations ROS generation ↑ (10 µM)	0.01 µM, 0.1 µM, 1 µM, 10 µM, 100 µM	Ghaemmaleki et al. [217]
		**INS-1** **cell line**	BPA	viability of cells after 48 h exposure ↓ (0.002 µM, 0.02 µM, 0.2 µM, 2 µM) insulin secretion with basal glucose concentration (5.6 mM) ↑ (0.02 µM), (−) (0.002 µM, 0.2 µM, 2 µM) insulin secretion with stimulatory glucose concentration (16.7 mM) ↑ (0.002 µM), ↓ (0.2 µM, 2 µM), (−) (0.02 µM) mRNA level of *insulin* ↓ (0.02 µM, 0.2 µM, 2 µM), (−) (0.002 µM) protein level of insulin ↓ (0.02 µM, 0.2 µM, 2 µM) mRNA level of genes involved in GSIS pathway: *GLUT2*, *GCK* ↓ (0.2 µM, 2 µM), (−) (0.002 µM, 0.02 µM; *KIR6.2*, *SUR* ↑ (0.002 µM), ↓ (0.02 µM, 0.2 µM, 2 µM) changes in mitochondrial morphology and mass ↑ (0.02 µM, 0.2 µM, 2 µM) cellular ATP level ↓ (0.02 µM, 0.2 µM, 2 µM), (−) (0.002 µM) mitochondrial potential ↓ (0.2 µM, 2 µM), (−) (0.002 µM, 0.02 µM) mRNA level of genes involved in mitochondrial metabolism and function: *UCP2* ↑ (0.02 µM, 0.2 µM, 2 µM), (−) (0.002 µM); *ATP6*, *Citrate synthase* ↓ (0.02 µM, 0.2 µM, 2 µM), (−) (0.002 µM); *TFAM* ↓ (0.2 µM, 2 µM), (−) (0.002 µM, 0.02 µM); *OGDH* ↓ (0.002 µM, 0.02 µM, 0.2 µM, 2 µM); *ND4L* (−) (0.002 µM, 0.02 µM, 0.2 µM, 2 µM) apoptosis ↑ (0.2 µM, 2 µM) release of cytochrome c from the mitochondria to the cytosol ↑ (0.02 µM, 0.2 µM, 2 µM), (−) (0.002 µM) protein level of pro-apoptotic protein BAX, APAF1, 17-kDa cleaved form of caspase-9 ↑ (0.02 µM, 0.2 µM, 2 µM), (−) (0.002 µM), 17-kDa form of cleaved caspase-3 ↑ (0.02 µM, 0.2 µM, 2 µM), (−) (0.002 µM), 19-kDa form of cleaved caspase-3 ↑ (0.002 µM, 0.02 µM, 0.2 µM, 2 µM), anti-apoptotic protein Bcl-2 ↓ (0.02 µM, 0.2 µM, 2 µM), (−) (0.002 µM), 40-kDa cleaved form of caspase-9 (−) (0.002 µM, 0.02 µM, 0.2 µM, 2 µM)	0.002 µM, 0.02 µM, 0.2 µM, 2 µM	Lin et al. [219]
		**INS-1E cell line**	BPA	cell viability after 48 h exposure ↓ (10 pM, 1 nM, 1 µM), (−) (0.1 pM, 1 pM, 100 pM) β-cell apoptosis ↑ (0.1 pM, 1 pM, 10 pM, 100 pM, 1 nM, 1 µM) ERα and ERβ involved in BPA-induced apoptosis ROS generation ↑ (1 nM, 1 µM) mRNA level of *MAFA*, *PDX1*, *INS1*, *INS2*, *GLUT2*, *GCK* (−) (0.1 pM, 1 pM, 10 pM, 100 pM, 1 nM, 1 µM)	0.1 pM, 1 pM, 10 pM, 100 pM, 1 nM, 1 µM	Dos Santos et al. [23]
			TBT	cell viability after 48 h exposure ↓ (20 nM, 50 nM, 100 nM, 200 nM), (−) (1 nM, 10 nM) β-cell apoptosis ↑ (10 nM, 20 nM, 50 nM, 100 nM, 200 nM), (−) (1 nM) PPARγ involved in TBT-induced apoptosis ROS generation ↑ (20 nM, 200 nM) mRNA level of *MAFA*, *PDX1* ↓ (200 nM), (−) (1 nM, 10 nM, 20 nM, 50 nM, 100 nM), *INS1*, *INS2*, *GLUT2*, *GCK* (−) (1 nM, 10 nM, 20 nM, 50 nM, 100 nM, 200 nM)	10 nM, 20 nM, 50 nM, 100 nM, 200 nM	Dos Santos et al. [23]
			PFOA	cell viability after 48 h exposure (−) (10 pM, 100 pM, 1 nM, 10 nM, 100 nM, 1 µM) β-cell apoptosisthe ↑ (10 µM, 20 µM, 50 µM, 10the 0 µM, 200 µM), (−) (1 nM, 1 µM) ROS generation (−) (1 nM, 1 µM) mRNA level of *MAFA*, *PDX1*, *INS1*, *INS2*, *GLUT2*, *GCK* (−) (1 nM, 1 µM)	10 µM, 20 µM, the 50 µM, 100 µM, 200 µM	Dos Santos et al. [23]
			TPP	cell viability after 48 h exposure (−) (10 pM, 100 the pM, 1 nM, 10 nM, 100 nM, 1 µM) β-cell apoptosis (−) (10 pM, 100 pM, 1 nM, 10 nM, 100 nM, 1 µM) mRNAthe level of *MAFA*, *PDX1*, *INS1*, *INS2*, *GLUT2*, *GCK* (−) (1 nM, 1 µM)	none of the the doses tested resulted in a biological effect	Dos Santos et al. [23]
			TCS	cell viability after 48 h exposure (−) (10 pM, 100 pM, 1 nM, 10 nM, 100 nM, 1 µM) β-cell apoptosis (−) (10 pM, 100 pM, 1 nM, 10 nM, 100 nM, 1 µM) mRNA level of *MAFA*, *PDX1*, *INS1*, *INS2*, *GLUT2*, *GCK* (−) (1 nM, 1 µM)	none of the doses tested resulted in a biological effect	Dos Santos et al. [23]
			DDE	cell viability after 48 h exposure (−) (10 pM, 100 pM, 1 nM, 10 nM, 100 nM, 1 µM) β-cell apoptosis (−) (10 pM, 100 pM, 1 nM, 10 nM, 100 nM, 1 µM) mRNA level of *MAFA*, *PDX1*, *INS1*, *INS2*, *GLUT2*, *GCK* (−) (1 nM, 1 µM)	none of the doses tested resulted in a biological effect	Dos Santos et al. [23]
			p,p’-DDE	protein expression of vitamin D-binding protein (VDBP) ↑, glucosidase 2 subunit beta precursor ↓ intracellular protein level of proinsulin, insulin monomer ↓, hexameric insulin (−) mRNA level of *INS1*, *INS2* ↓, *VDBP* (−)	10 µM	Pavlíková et al. [220]
			p,p’-DDT	protein expression of tubulin beta-5 chain, annexin A4, vitamin D-binding protein (VDBP) ↑, actin, mortalin/GRP75 ↓ intracellular protein level of proinsulin, hexameric insulin, insulin monomer ↓ mRNA level of *VDBP* ↑, *INS1*, *INS2* ↓	10 µM	Pavlíková et al. [220]
		**RIN-m5F cell line**	TBT	viability of cells (−) (0.05 µM, 0.1 µM, 0.2 µM) GSIS (20 mM glucose) ↑ (0.1 µM, 0.2 µM), (−) (0.05 µM) insulin secretion ↑ (0.1 µM) intracellular calcium level ↑ (0.1 µM, 0.2 µM) protein level of p-PKC, p-ERK1/2 ↑, p-Akt (−) (0.1 µM, 0.2 µM) level of intracellular ROS ↑ (0.05 µM, 0.1 µM, 0.2 µM)	0.05 µM, 0.1 µM, 0.2 µM	Chen et al. [106]
				viability of cells ↓ (0.5 µM, 1 µM), (−) (0.1 µM, 0.2 µM) GSIS (after 24 h exposure) ↓ (0.5 µM, 1 µM) apoptosis ↑ (0.5 µM) cleavage of PARP ↑ (0.5 µM, 1 µM) level of p-JNK, p-ERK1/2 ↑, p-38 (−) (0.5 µM, 1 µM) intracellular ROS generation ↑ (0.2 µM, 0.5 µM, 1 µM) caspase-3 activity ↑ (0.5 µM)	0.2 µM, 0.5 µM, 1 µM	Huang et al. [221]
			PFOA	viability of cells ↓ (100 µM, 200 µM, 300 µM, 500 µM), (−) (1 µM) apoptosis ↑ (100 µM, 300 µM, 500 µM), (−) (1 µM) ROS generation ↑ (200 µM, 300 µM, 400 µM, 500 µM), (−) (1 µM, 10 µM, 50 µM, 100 µM) mitochondrial superoxide accumulation ↑ (200 µM, 300 µM, 500 µM), (−) (1 µM, 10 µM, 50 µM, 100 µM) NO (nitric oxide) production ↑ (50 µM, 100 µM, 200 µM, 300 µM, 400 µM, 500 µM), (−) (1 µM, 10 µM) cytosolic level of TNFα ↑ (100 µM, 150 µM, 200 µM, 300 µM, 500 µM), (−) (10 µM), Il1β ↑ (200 µM, 300 µM, 500 µM), (−) (10 µM, 100 µM, 150 µM) MMP collapse ↑ (300 µM, 500 µM), (−) (1 µM, 10 µM, 50 µM, 100 µM, 200 µM) intracellular ATP level ↑ (10 µM, 50 µM), ↓ (200 µM, 300 µM, 500 µM), (−) (1 µM, 100 µM) cytochrome c release ↑ (200 µM, 300 µM, 500 µM), (−) (10 µM, 100 µM) cardiolipin peroxidation ↑ (200 µM, 300 µM, 500 µM), (−) (10 µM, 100 µM)	10 µM, 50 µM, 100 µM, 150 µM, 200 µM, 300 µM, 400 µM, 500 µM	Suh et al. [222]
		**Primary β-cells from wild type mouse**	BPA	β-cell apoptosis ↑ (1 nM, 1 µM)	1 nM, 1 µM	Dos Santos et al. [23]
				insulin secretion in the presence of 8 mM glucose ↑ K_ATP_ channel activity ↓ frequency of [Ca^2+^]_i_ oscillations ↑	1 nM	Soriano et al. [223]
				viability of cells (after 48 h exposure) ↓ (0.001 µM, 1 µM, 100 µM) number of apoptotic cells (after 48 h exposure) ↑ (0.001 µM, 1 µM, 100 µM) mRNA level of genes encoding components of respiratory chain complexes: *NDUFS4*, *UQCRB*, genes involved in ATP production and/or in insulin exocytosis process: *VAPA*, *ATP1B1*, *ATP6V1F*, genes involved in detoxification: *SOD2*, *GPX3*, *ZFAND2A*, anti-apoptotic gene *BCL-2* ↓ (0.001 µM), pro-apoptotic gene Bax ↑ (0.001 µM) ROS level ↑ (0.001 µM) MMP ↓ (0.001 µM) GSIS (16 mM glucose) ↓ (0.001 µM, 100 µM) probable mechanism of action via activation of NF-kB pathway	0.001 µM, 1 µM, 100 µM	Carchia et al. [105]
			BPS	insulin secretion in response to 16.7 mM concentration (during 1 h treatment of BPS) ↑ (1 nM, 1 µM) insulin secretion in response to 8.3 mM concentration (during 48 h treatment of BPS) ↑ (1 nM, 1 µM) insulin secretion in response to 16.7 mM concentration (during 48 h treatment of BPS) ↑ (1 nM), (−) (1 µM) K_ATP_ channel activity ↓ (1 nM) mRNA level of (after 48 h treatment of BPS) *CACNA1E*, *KCNMa1*, *SCN9A* ↓ (1 nM), (−) (100 nM, 1 µM), *KCNIP* ↑ (1 nM), (−) (100 nM, 1 µM) Ca^2+^ currents ↓ (1 nM), (−) (100 nM, 1 µM)	1 nM, 1 µM	Marroqui et al. [224]
			BPF	insulin secretion in response to 16.7 mM concentration (during 1 h treatment of BPF) ↑ (1 nM, 1 µM) insulin secretion in response to 16.7 mM concentration (during 48 h treatment of BPF) ↑ (1 µM) K_ATP_ channel activity ↓ (10 nM), (−) (1 nM) mRNA level of (after 48 h treatment of BPF) *CACNA1E* ↓ (100 nM, 1 µM), (−) (1 nM), *KCNMA1*, *SCN9A*, *KCNIP1* ↓ (1 µM), (−) (1 nM, 100 nM) Ca^2+^ currents ↓ (1 µM), (−) (1 nM, 100 nM)	1 nM, 10 nM, 100 nM, 1 µM	Marroqui et al. [224]
			TBT	β-cell apoptosis ↑ (20 nM, 200 nM)	20 nM, 200 nM	Dos Santos et al. [23]
				GSIS (20 mM glucose) ↑ ROS generation ↑	0.1 µM, 0.2 µM	Chen et al. [106]
			PFOA	β-cell apoptosis (−) (1 nM, 1 µM)	none of the doses tested resulted in a biological effect	Dos Santos et al. [23]
			TPP	β-cell apoptosis (−) (1 nM, 1 µM)	none of the doses tested resulted in a biological effect	Dos Santos et al. [23]
			TCS	β-cell apoptosis (−) (1 nM, 1 µM)	none of the doses tested resulted in a biological effect	Dos Santos et al. [23]
			DDE	β-cell apoptosis (−) (1 nM, 1 µM)	none of the doses tested resulted in a biological effect	Dos Santos et al. [23]
		**Primary β-cells from ERβ-/- mouse (estrogen receptor β (ERβ) knockout (BERKO) mouse)**	BPA	insulin secretion in the presence of 8 mM glucose (−) K_ATP_ channel activity (−) frequency of [Ca^2+^]_i_ oscillations ↓	1 nM	Soriano et al. [223]
			BPS	K_ATP_ channel activity (−) (1 nM) Ca^2+^ currents (−) (1 nM, 100 nM, 1 µM) mRNA level of (after 48 h treatment of BPS) *CACNA1E*, *KCNMA1*, *SCN9A* (−) (1 nM)	none of the doses tested resulted in a biological effect	Marroqui et al. [224]
			BPF	K_ATP_ channel activity (−) (1 nM, 10 nM) Ca^2+^ currents (−) (1 nM, 100 nM, 1 µM) mRNA level of (after 48 h treatment of BPF) *CACNA1E* ↓, *KCNMA1*, *SCN9A* (−) (1 µM)	1 µM	Marroqui et al. [224]
		**MIN-6 cell line**	BPA	viability of cells (after 24 h exposure) ↓ (100 pM, 1 nM, 10 nM, 100 nM, 1 µM), (−) (10 µM) mRNA level of *PDX1* ↑ (100 pM, 10 nM, 1 µM), (−) (1 nM, 100 nM, 10 µM); *HNF4α* ↑ (100 pM, 10 nM, 1 µM, 10 µM), (−) (1 nM, 100 nM); *KIR6.2* ↑ (10 nM), (−) (100 pM, 1 nM, 100 nM, 1 µM, 10 µM); *MAFA* ↑ (100 nM), (−) (100 pM, 1 nM, 10 nM, 1 µM, 10 µM); *INS*, *SUR1*, *GLUT2*, *GCK* (−) (100 pM, 1 nM, 10 nM, 100 nM, 1 µM, 10 µM) GSIS (20 mM glucose) ↑ (100 nM), (−) (100 pM, 1 nM, 10 nM, 1 µM, 10 µM) insulin secretion in response to low glucose concentration (1.67 mM) ↑ (100 nM, 10 µM), (−) (100 pM, 1 nM, 10 nM, 1 µM) insulin content ↑ (10 µM), (−) (100 pM, 1 nM, 10 nM, 100 nM, 1 µM)	100 pM, 1 nM, 10 nM, 100 nM, 1 µM, 10 µM	Al-Abdulla et al. [229]
			BPS	viability of cells (after 24 h exposure) ↓ (100 pM, 1 nM, 10 nM, 100 nM, 1 µM, 10 µM) mRNA level of *MAFA* ↑ (10 nM, 100 nM), (−) (100 pM, 1 nM, 1 µM, 10 µM); *KIR6.2* ↑ (10 nM, 100 nM, 10 µM), (−) (100 pM, 1 nM, 1 µM); *HNF4α* ↓ (100 pM), (−) (1 nM, 10 nM, 100 nM, 1 µM, 10 µM); *GlUT2* ↓ (1 nM, 10 nM, 100 nM, 1 µM), (−) (100 pM, 10 µM), *INS*, *PDX1*, *SUR1*, *GCK* (−) (100 pM, 1 nM, 10 nM, 100 nM, 1 µM, 10 µM) GSIS (20 mM) ↓ (100 pM, 10 nM, 1 µM, 10 µM), (−) (1 nM, 100 nM) insulin secretion in response to low glucose concentration (1.67 mM) (−) (100 pM, 1 nM, 10 nM, 100 nM, 1 µM, 10 µM) insulin content ↓ (10 nM, 100 nM, 1 µM, 10 µM), (−) (100 pM, 1 nM)	100 pM, 1 nM, 10 nM, 100 nM, 1 µM, 10 µM	Al-Abdulla et al. [229]
			DEHP	viability of cells (after 24 h exposure) ↓ (1 µM), (−) (100 pM, 1 nM, 10 nM, 100 nM, 10 µM) mRNA level of *SUR1*, *GLUT2* ↑ (10 µM), (−) (100 pM, 1 nM, 10 nM, 100 nM, 1 µM); *INS*, *PDX1*, *HNF4α*, *MAFA*, *KIR6.2*, *GCK* (−) (100 pM, 1 nM, 10 nM, 100 nM, 1 µM, 10 µM) GSIS (20 mM glucose) ↓ (100 pM, 1 nM, 10 nM, 100 nM, 1 µM, 10 µM) insulin secretion in response to low glucose concentration (1.67 mM) (−) (100 pM, 1 nM, 10 nM, 100 nM, 1 µM, 10 µM) insulin content ↓ (1 µM), (−) (100 pM, 1 nM, 10 nM, 100 nM, 10 µM)	100 pM, 1 nM, 10 nM, 100 nM, 1 µM, 10 µM	Al-Abdulla et al. [229]
			PFOS	viability of cells (after 24 h exposure) ↓ (100 pM, 1 nM, 10 nM, 100 nM, 1 µM, 10 µM) mRNA level of *INS* ↑ (1 nM), (−) (100 pM, 10 nM, 100 nM, 1 µM, 10 µM), *HNF4α* ↑ (10 nM), (−) (100 pM, 1 nM, 100 nM, 1 µM, 10 µM), *MAFA* ↓ (1 µM), (−) (100 pM, 1 nM, 10 nM, 100 nM, 10 µM), *GLUT2* ↓ (10 nM, 100 nM, 1 µM, 10 µM), (−) (100 pM, 1 nM), *PDX1*, *KIR6.2*, *SUR1*, *GCK* (−) (100 pM, 1 nM, 10 nM, 100 nM, 1 µM, 10 µM) GSIS (20 mM) ↓ (100 pM, 100 nM, 10 µM), (−) (1 nM, 10 nM, 1 µM) insulin secretion in response to low glucose concentration (1.67 mM) (−) (100 pM, 1 nM, 10 nM, 100 nM, 1 µM, 10 µM) insulin content (−) (100 pM, 1 nM, 10 nM, 100 nM, 1 µM, 10 µM)	100 pM, 1 nM, 10 nM, 100 nM, 1 µM, 10 µM	Al-Abdulla et al. [229]
			CdCl_2_	viability of cells (after 24 h exposure) ↓ (100 pM, 1 nM, 10 nM, 100 nM, 1 µM, 10 µM) mRNA level of *INS* ↑ (100 pM, 100 nM), (−) (1 nM, 10 nM); *MAFA* ↑ (100 nM), (−) (100 pM, 1 nM, 10 nM); *PDX1*, *HNF4α*, *KIR6.2*, *SUR1*, *GLUT2*, *GCK* (−) (100 pM, 1 nM, 10 nM, 100 nM) GSIS (20 mM glucose) (−) (100 pM, 1 nM, 10 nM, 100 nM, 1 µM) insulin secretion in response to low glucose concentration (1.67 mM) ↓ (100 pM), (−) (1 nM, 10 nM, 100 nM, 1 µM) insulin content (−) (100 pM, 1 nM, 10 nM, 100 nM, 1 µM)	100 pM, 1 nM, 10 nM, 100 nM, 1 µM, 10 µM	Al-Abdulla et al. [229]
			DDE	viability of cells (after 24 h exposure) ↓ (10 nM), (−) (100 pM, 1 nM, 100 nM, 1 µM, 10 µM) mRNA level of *SUR1* ↑ (100 pM), (−) (1 nM, 10 nM, 100 nM, 1 µM, 10 µM); *INS* ↓ (1 nM, 1 µM, 10 µM), (−) (100 pM, 10 nM, 100 nM); *MAFA* ↓ (1 nM), (−) (1 nM, 10 nM, 100 nM, 1 µM, 10 µM); *PDX1; HNF4α*, *KIR6.2*, *GLUT2*, *GCK* (−) (100 pM, 1 nM, 10 nM, 100 nM, 1 µM, 10 µM) GSIS (20 mM) (−) (100 pM, 1 nM, 10 nM, 100 nM, 1 µM, 10 µM) insulin secretion in response to low glucose concentration (1.67 mM) (−) (100 pM, 1 nM, 10 nM, 100 nM, 1 µM, 10 µM) insulin content (−) (100 pM, 1 nM, 10 nM, 100 nM, 1 µM, 10 µM)	100 pM, 1 nM, 10 nM, 1 µM, 10 µM	Al-Abdulla et al. [229]
		**β-TC-6 cell line**	PFOS	GSIS (1.4 mM glucose) ↑ (50 µM, 100 µM) insulin secretion in the absence of glucose ↑ (50 µM) intracellular calcium level ↑ (5 µM, 10 µM, 50 µM, 100 µM) probable mechanism of action via GPR40 activation	5 µM, 10 µM, 50 µM, 100 µM	Qin et al. [231]
			p,p’-DDE	basal insulin secretion ↑ (10 µM) GSIS (5 mM) ↑ (10 µM) ROS level (−) (10 µM) intracellular protein level of *PDX1* (−) (1 µM, 10 µM, 100 µM) PC enzyme activity ↑ (10 µM), (−) (100 µM)	10 µM	Ward et al. [232]
	**Human**	**Pancreatic islets**	BPA	insulin secretion in the presence of 8 mM glucose ↑ K_ATP_ channel activity ↓	1 nM	Soriano et al. [223]
			TBT	GSIS (20 mM glucose) ↑	0.1 µM	Chen et al. [106]
		**EndoC-βH1 cell line**	BPA	cell viability after 48 h exposure ↓ (1 pM, 10 pM, 100 pM, 1 nM, 1 µM), (−) (0.1 pM) β-cell apoptosis ↑ (1 pM, 10 pM, 100 pM, 1 nM, 1 µM), (−) (0.1 pM) caspase 3/7 activity ↑ (10 nM, 1 µM) Erα and Erβ involved in BPA-induced apoptosis ROS generation ↑ (1 nM, 1 µM) GSIS (20 mM glucose) (−) (0.1 pM, 1 pM, 10 pM, 100 pM, 1 nM, 1 µM) insulin content (−) (0.1 pM, 1 pM, 10 pM, 100 pM, 1 nM, 1 µM) mRNA level of *MAFA*, *PDX1*, *INS*, *GLUT2*, *GCK* (−) (0.1 pM, 1 pM, 10 pM, 100 pM, 1 nM, 1 µM)	1 pM, 10 pM, 100 pM, 1 nM, 10 nM, 1 µM	Dos Santos et al. [23]
				cell viability after 72 h exposure (−) (1 nM, 10 nM, 100 nM, 1 µM) mRNA level of *MAFB* ↑ (10 nM), (−) (1 nM, 100 nM), *SNAP25* ↑ (1 nM), (−) (10 nM, 100 nM), *INS*, *PDX1*, *HNF4α*, *MAFA*, *KIR6.2*, *SUR1*, *GLUT1*, *GCK* (−) (1 nM, 10 nM, 100 nM, 1 µM) GSIS (20 mM glucose) ↑ (10 nM, 100 nM, 1 µM), (−) (1 nM) basal insulin secretion ↑ (1 µM), (−) (1 nM, 10 nM, 100 nM) insulin content ↑ (1 nM, 10 nM, 100 nM, 1 µM)	1 nM, 10 nM, 100 nM, 1 µM	Al-Abdulla et al. [229]
			BPS	cell viability after 48 h exposure ↓ (1 µM), (−) (1 nM, 10 nM, 100 nM) mRNA level of *HNF4α* ↑ (1 nM), (−) (10 nM, 100 nM), *MAFA* ↓ (100 nM), (−) (1 nM, 10 nM), *MAFB*, *GLUT1* ↓ (10 nM, 100 nM), (−) (1 nM), *KIR6.2* ↓ (10 nM), (−) (1 nM, 100 nM), *SNAP25* ↓ (1 nM), (−) (10 nM, 100 nM), *INS*, *PDX1*, *SUR1*, *GCK* (−) (1 nM, 10 nM, 100 nM) GSIS (20 mM) ↓ (1 nM, 1 µM), (−) (10 nM, 100 nM) basal insulin secretion (−) (1 nM, 10 nM, 100 nM, 1 µM) insulin content (−) (1 nM, 10 nM, 100 nM, 1 µM)	1 nM, 10 nM, 100 nM, 1 µM	Al-Abdulla et al. [229]
			BPF	cell viability after 72 h exposure ↓ (1 µM), (−) (1 nM, 10 nM, 100 nM) GSIS (20 mM glucose) (−) (1 nM, 10 nM, 100 nM, 1 µM) insulin secretion in response to low glucose concentration (2.8 mM) (−) (1 nM, 10 nM, 100 nM, 1 µM)	1 µM	Al-Abdulla et al. [229]
			DEHP	cell viability after 7 days of exposure ↓ (1 nM, 10 nM, 100 nM), (−) (1 µM) mRNA level of *INS*, *PDX1*, *HNF4α*, *MAFA*, *MAFB*, *KIR6.2*, *SUR1*, *SNAP25*, *GLUT1*, *GCK* (−) (1 nM, 10 nM, 100 nM) GSIS (20 mM) ↑ (1 µM), ↓ (1 nM, 10 nM, 100 nM) insulin secretion in response to low glucose concentration (2.8 mM) (−) (1 nM, 10 nM, 100 nM, 1 µM) insulin content ↑ (1 µM), ↓ (1 nM, 10 nM, 100 nM)	1 nM, 10 nM, 100 nM, 1 µM	Al-Abdulla et al. [229]
			TBT	cell viability after 48 h exposure ↓ (20 nM, 50 nM, 100 nM, 200 nM), (−) (1 nM, 10 nM) β-cell apoptosis ↑ (1 nM, 20 nM, 50 nM, 100 nM, 200 nM), (−) (10 nM) caspase 3/7 activity ↑ (20 nM, 200 nM) PPARγ involved in TBT-induced apoptosis ROS generation ↑ (20 nM, 200 nM) GSIS ↑ (20 mM glucose), (−) (1 nM, 10 nM, 20 nM, 50 nM, 100 nM) insulin content ↑ (20 nM), ↓ (200 nM), (−) (1 nM, 10 nM, 50 nM, 100 nM) mRNA level of *GLUT2* ↑ (200 nM), (−) (1 nM, 10 nM, 20 nM, 50 nM, 100 nM), *MAFA* ↓ (10 nM, 20 nM, 50 nM, 100 nM, 200 nM), (−) (1 nM), *PDX1*, *INS*, *GCK* (−) (1 nM, 10 nM, 20 nM, 50 nM, 100 nM, 200 nM)	1 nM, 20 nM, 50 nM, 100 nM, 200 nM	Dos Santos et al. [23]
			PFOA	cell viability after 48 h exposure (−) (10 pM, 100 pM, 1 nM, 10 nM, 100 nM, 1 µM) β-cell apoptosis ↑ (20 µM, 50 µM, 100 µM, 200 µM), (−) (1 nM, 1 µM) caspase 3/7 activity (−) (1 µM) ROS generation (−) (1 nM, 1 µM) GSIS (20 mM glucose) ↓ (10 pM, 1 nM, 10 nM, 100 nM), (−) (100 pM, 1 µM) insulin secretion at low glucose concentration (0 mM) ↓ (1 nM), (−) (10 pM, 100 pM, 10 nM, 100 nM, 1 µM) insulin content (−) (10 pM, 100 pM, 1 nM, 10 nM, 100 nM, 1 µM) mRNA level of *MAFA*, *PDX1*, *INS*, *GLUT2*, *GCK* (−) (1 nM, 1 µM)	10 pM, 1 nM, 10 nM, 100 nM, 20 µM, 50 µM, 100 µM, 200 µM	Dos Santos et al. [23]
			PFOS	cell viability after 72 h exposure (−) (1 nM, 10 nM, 100 nM, 1 µM) mRNA level of *INS*, *PDX1*, *HNF4α*, *MAFA*, *MAFB*, *KIR6.2*, *SUR1*, *SNAP25*, *GLUT1*, *GCK* (−) (10 nM, 100 nM) GSIS (20 mM glucose) ↑ (10 nM, 100 nM), (−) (1 nM, 1 µM) insulin secretion in response to low glucose concentration (2.8 mM) ↑ (10 nM, 1 µM), (−) (1 nM, 100 nM) insulin content ↑ (1 nM, 10 nM, 1 µM), (−) (100 nM)	1 nM, 10 nM, 100 nM, 1 µM	Al-Abdulla et al. [229]
			TPP	cell viability after 48 h exposure (−) (10 pM, 100 pM, 1 nM, 10 nM, 100 nM, 1 µM) β-cell apoptosis (−) (1 nM, 1 µM) caspase 3/7 activity (−) (1 µM) GSIS (20 mM glucose) ↑ (1 µM), (−) (10 pM, 100 pM, 1 nM, 10 nM, 100 nM) insulin secretion at low glucose concentration (0 mM) ↑ (100 pM) insulin content (−) (10 pM, 100 pM, 1 nM, 10 nM, 100 nM, 1 µM) mRNA level of *MAFA*, *PDX1*, *INS*, *GLUT2*, *GCK* (−) (1 nM, 1 µM)	100 pM, 1 µM	Dos Santos et al. [23]
			TCS	cell viability after 48 h exposure (−) (10 pM, 100 pM, 1 nM, 10 nM, 100 nM, 1 µM) β-cell apoptosis (−) (1 nM, 1 µM) caspase 3/7 activity (−) (1 µM) GSIS (−) (10 pM, 100 pM, 1 nM, 10 nM, 100 nM, 1 µM) insulin content (−) (10 pM, 100 pM, 1 nM, 10 nM, 100 nM, 1 µM) mRNA level of *MAFA*, *PDX1*, *INS*, *GLUT2*, *GCK* (−) (1 nM, 1 µM)	none of the doses tested resulted in a biological effect	Dos Santos et al. [23]
			CdCl_2_	cell viability after 72 h exposure ↓ (1 nM, 10 nM, 100 nM), (−) (1 µM) mRNA level of *HNF4α*, *SNAP25* ↓ (100 nM), (−) (10 nM), *INS*, *PDX1*, *MAFA*, *MAFB*, *KIR6.2*, *SUR1*, *GLUT1*, *GCK* (−) (10 nM, 100 nM) GSIS (20 mM glucose) ↑ (10 nM), (−) (1 nM, 100 nM, 1 µM) insulin secretion in response to low glucose concentration (2.8 mM) (−) (1 nM, 10 nM, 100 nM, 1 µM) insulin content (−) (1 nM, 10 nM, 100 nM, 1 µM)	1 nM, 10 nM, 100 nM	Al-Abdulla et al. [229]
			DDE	cell viability after 48 h exposure (−) (10 pM, 100 pM, 1 nM, 10 nM, 100 nM, 1 µM) β-cell apoptosis (−) (1 nM, 1 µM) caspase 3/7 activity (−) (1 µM) GSIS (20 mM glucose) ↑ (10 nM, 1 µM), (−) (10 pM, 100 pM, 1 nM, 100 nM) insulin content (−) (10 pM, 100 pM, 1 nM, 10 nM, 100 nM, 1 µM) mRNA level of *MAFA*, *PDX1*, *INS*, *GLUT2*, *GCK* (−) (1 nM, 1 µM)	10 nM, 1 µM	Dos Santos et al. [23]
				cell viability after 7 days of exposure ↓ (1 nM, 10 nM, 100 nM, 1 µM) mRNA level of *INS*, *PDX1*, *HNF4α*, *MAFA*, *MAFB*, *KIR6.2*, *SUR1*, *SNAP25*, *GLUT1*, *GCK* (−) (1 nM, 10 nM, 100 nM) GSIS (20 mM glucose) (−) (1 nM, 10 nM, 100 nM, 1 µM) insulin secretion in response to low glucose concentration (2.8 mM) (−) (1 nM, 10 nM, 100 nM, 1 µM) insulin content (−) (1 nM, 10 nM, 100 nM, 1 µM)	1 nM, 10 nM, 100 nM, 1 µM	Al-Abdulla et al. [229]
		**NES2Y cell line**	p,p’-DDT	viability of cells after 24 h and 48 h exposure ↓ (100 µM), (−) (0.1 nM, 1 nM, 10 nM, 0.1 µM, 1 µM, 10 µM) protein expression of cytokeratin 8, cytokeratin 18, alpha-enolase, actin ↓ (10 µM)	10 µM, 100 µM	Pavlikova et al. [234]
				viability of cells after 24 h exposure ↓ (150 µM, 175 µM, 200 µM), (−) (100 µM, 125 µM) protein expression of cleaved caspase -6, -7, -8, -9, cleaved PARP, CHOP, GRP78 (BiP), GRP75 (mortalin), NDRG1, EFHD2 ↑, ECHM, vimentin, HSP27, IDH3A, K2C8, HNRPF, BIEA, EF2, EZRI, FRIL, G3BP1, HNRH1, NDUS3, NDUS1, PBDC1, PCNA, TCPA ↓ (150 µM)	150 µM, 175 µM, 200 µM	Pavlikova et al. [107]
			p,p’-DDE	viability of cells after 24 h and 48 h exposure ↓ (100 µM), (−) (0.1 nM, 1 nM, 10 nM, 0.1 µM, 1 µM, 10 µM) protein expression of cytokeratin 18, HNRH1 ↓ (10 µM)	10 µM, 100 µM	Pavlikova et al. [234]
		**PANC-1 cell line**	BPA	mRNA level of *PCSK1* ↓, insulin secretion ↓	10 nM	Menale et al. [124]

Legend: ↑ increase; ↓ decrease; (−) no observed effects; * concentration(s) at which biological effects were observed.

## Data Availability

Not applicable.

## Abbreviations

1,3-DCP	1,3-dichloro-2-propanol
11β-HSD1	11β-hydroxysteroid dehydrogenase type 1
4-HP	4-hexylphenol
4-NP	4-nonylphenol
AACS	acetoacetyl-CoA synthetase
ABCA1	ATP-binding cassette transporter A1
ACACA	acetyl-CoA carboxylase α
ACAT3	acetyl-coenzyme A acetyltransferase 3
ACC	acetyl-CoA carboxylase
ACC2	acetyl-CoA carboxylase 2
ACLY	adenosine triphosphate citrate lyase
ACTB	actin
Act-D	actinomycin-D
ADIPOQ	adiponectin, C1Q and collagen domain containing
ADIPOR2	adiponectin receptor 2
ADP	adenosine diphosphate
ADSCs	animal adipose-derived stem cells
AEA	anandamide (N-arachidonoylethanolamine)
AKT2	v-akt murine thymoma viral oncogene homolog 2
ALT	alanine aminotransferase
AML12	alpha mouse liver 12
AMP	adenosine monophosphate
AMPK	AMP-activated protein kinase
AMPKα	AMP-activated protein kinase-α
AOX	acyl-CoA oxidase
AP2	adipocyte protein 2
APAF1	apoptotic protease activating factor 1
APM1	adipocyte most abundant gene transcript-1
APOA1BP	apolipoprotein A1-binding protein
APOA2	apolipoprotein A2
APOA4	apolipoprotein A4
APOA-I	apolipoprotein A-I
APOB	apolipoprotein B
APOE	apolipoprotein E
ASCs	adipose-derived stem cells
AST	aspartate aminotransferase
AT	adipose tissue
ATGL	adipose triglyceride lipase
ATP	adenosine triphosphate
ATP1B1	ATPase Na+/K+ transporting subunit beta 1
ATP6	ATP synthase subunit 6
ATP6v1F	ATPase H+ transporting V1 subunit F
BADGE	2HCl bisphenol A bis(3-chloro-2-hydroxypropyl) ether
BAT	brown adipose tissue
BAX	BCL2 associated X apoptosis regulator
BBP	benzyl butyl phthalate
BCL-2	apoptosis regulator Bcl-2
BHPF	fluorene-9-bisphenol
BIEA	biliverdin reductase
BMI	body mass index
BMMSC	bone marrow-derived mesenchymal stem cells
BPA	bisphenol A
BPAF	bisphenol AF
BPB	bisphenol B
BPF	bisphenol F
BPS	bisphenol S
C/EBP	CCAAT/enhancer-binding protein
C/EBPα	CCAAT/enhancer-binding protein α
C/EBPβ	CCAAT/enhancer-binding protein β
C/EBPδ	CCAAT/enhancer-binding protein δ
CACNA1E	calcium voltage-gated channel subunit alpha1 E
CaMKII	Ca^2+^/calmodulin-dependent protein kinase II
cAMP	cyclic adenosine monophosphate
CASP3B	caspase 3B
CAT	catalase
CB1	endocannabinoid receptor type 1
CBS	cystathionine-beta-synthase
CCL20	chemokine (C-C motif) ligand 20
Cd	cadmium
CD36	fatty acid translocase
CdCl_2_	cadmium chloride
CDKN1B	cyclin-dependent kinase inhibitor 1B
Cer	ceramide
CHOP	C/EBP homologous protein
CIDEA	cell death-inducing DNA fragmentation factor-alpha-like effector A
CK18	cytokeratin 18
CK8	cytokeratin 8
CNR1	cannabinoid receptor 1
COX16	cytochrome C oxidase assembly factor
CPF	chlorpyrifos
CPT1	carnitine palmitoyltransferase 1
CPT1A	carnitine palmitoyltransferase 1 a
CPT1B	carnitine palmitoyltransferase 1 b
CREB	cAMP response element-binding protein
CYP1A1	cytochrome P450 family 1 subfamily A member 1
CYP2C18	cytochrome P450 family 2 subfamily C member 18
CYP2C19	cytochrome P450 family 2 subfamily C member 19
CYP2C9	cytochrome P450 family 2 subfamily C member 9
CYP3A	cytochrome P450 family 3 subfamily A
CYP3A4	cytochrome P450 family 3 subfamily A member 4
CYP3A65	cytochrome P450 family 3 subfamily A polypeptide 65
CYPs	cytochromes
D2EFHD2	EF-hand domain-containing protein
DBP	dibutyl phthalate
DCHP	dicyclohexyl phthalate
DDE	dichlorodiphenyldichloroethylene
DDT	dichlorodiphenyltrichloroethane
DEHP	di(2-ethylhexyl) phthalate
DEX	dexamethasone
DG	diacylglycerol
DGAT1	diacylglycerol acyltransferase 1
DGAT1A	diacylglycerol acyltransferase 1a
DGAT2	diacylglycerol acyltransferase 2
DIDP	diisodecyl phthalate
DINCH	bis(7-methyloctyl) cyclohexane-1,2-dicarboxylate
DINP	diisononyl phthalate
DLK	dual leucine zipper-bearing kinase
DNMT1	DNA (cytosine-5-)-methyltransferase 1
DNMT3A	DNA methyltransferase 3 alpha
DNMT3AA	DNA (cytosine-5-)-methyltransferase 3A
DNMT3B	DNA methyltransferase 3 beta
DPHP	bis(2-propylheptyl) phthalate
ECHM	enoyl-CoA hydratase
EDCs	endocrine-disrupting chemicals
EF2	elongation factor 2
EFHD2	EF-hand domain family member D2
ELOVL6	ELOVL fatty acid elongase 6
ENO1	α-enolase
ER	stress endoplasmic reticulum stress
ERK1/2	extracellular signal-regulated protein kinase 1/2
ERRγ	estrogen-related receptor γ
ERs	estrogen receptors
ERα	estrogen receptor α
ERβ -/- (or BERKO) mice	estrogen receptor β knockout mice
ERβ	estrogen receptor β
ESR1	estrogen receptor 1
ESR2	estrogen receptor 2
ESRRA	estrogen related receptor alpha
ether-TGs	ether-triacylglycerides
EU	European Union
Eurostat	Statistical Office of the European Union
EZRI	ezrin
FA	fatty acid
FAAH	fatty acid amide hydrolase
FABP	fatty acid binding protein
FABP4	fatty acid binding protein 4
FABP5	fatty acid binding protein 5
FADS1	fatty acid desaturase 1
FADS2	fatty acid desaturase
FAS	fatty acid synthase
FASN	fatty acid synthase
FATP1	fatty acid transport protein 1
FOS	Fos proto-oncogene, AP-1 transcription factor subunit
FOXO1	forkhead box protein O1
FRIL	ferritin light chain
FSP27	fat-specific protein 27
G3BP1	Ras GTPase-activating protein-binding 1
GCK	glucokinase
GDF15	growth differentiation factor 15
GLU2β	glucosidase 2 subunit beta
GLUT1	glucose transporter type 1
GLUT2	glucose transporter type 2
GLUT4	glucose transporter type 4
GLY	glyphosate
GPAT	glycerol-3-phosphate acyltransferase
GPAT	glycerol-3-phosphate acyltransferase
GPAT3	glycerol-3-phosphate acyltransferase 3
GPD1	glycerol-3-phosphate-dehydrogenase
GPR109B	protein-coupled receptor 109B
GPR30	G protein-coupled receptor 30
GPR40	G protein-coupled receptor 40
GPR41	G protein-coupled receptor 41
GPR43	G protein-coupled receptor 43
GPX1	glutathione peroxidase 1
GPX3	glutathione peroxidase 3
GPX4	glutathione peroxidase 4
GPX8	glutathione peroxidase 8
GR	glucocorticoid receptor
GRP75	75 kDa glucose-regulated protein
GRP78	78 kDa glucose-regulated protein
GSH	glutathione
GSIS	glucose-stimulated insulin secretion
GSR	glutathione-disulfide reductase
GSSG	oxidized glutathione
GSTA1/2	glutathione S-transferase alpha ½
GSTA3	glutathione S-transferase alpha 3
GSTO1	glutathione S-transferase omega-1
GUSB	glucuronidase beta
hADSCs	human adipose-derived stem cells
HBCD	hexabromocyclododecane
HL	hepatic lipase
HMGCR	3-hydroxy-3-methylglutaryl CoA reductase
HMOX1	heme oxygenase 1
HNF1B	hepatocyte nuclear factor 1b
HNF4α	hepatocyte nuclear factor 4 alpha
HNRH1	heterogenous nuclear ribonucleoprotein H1
HNRPF	heterogeneous nuclear ribonucleoprotein F
HSD11B1	11beta-hydroxysteroid dehydrogenase 1
HSL	hormone-sensitive lipase
HSP27	heat shock protein 27
HSPA1A	heat shock protein family A (Hsp70) member 1A
HSPA8	heat shock protein family A (Hsp70) member 8
hTERT	human telomerase reverse transcriptase
IBMX	phosphodiesterase inhibitor 1-methyl-3-isobutyl xanthine
IDH3A	isocitrate dehydrogenase (NAD(+)) 3 catalytic subunit alpha
IFN-γ	interferon gamma
IGF1	insulin-like growth factor 1
IL18	interleukin 18
IL1B	(IL1β) interleukin 1 beta
IL1α	interleukin 1 alpha
IL6	interleukin 6
INS1	insulin 1
INS2	insulin 2
INSIG1	insulin induced gene 1
INSR	insulin receptor
IR	insulin resistance
IRS-1	insulin receptor substrate 1
IRS-2	insulin receptor substrate 2
IR-β	insulin receptor subunit β
JAK2	Janus kinase 2
JNK	c-Jun N-terminal kinase
K2C8	keratin type II cytoskeletal 8
K_ATP_	channel ATP-sensitive K^+^ channel
KCNIP	potassium voltage-gated channel interacting protein
KCNIP1	potassium voltage-gated channel interacting protein 1
KCNMA1	potassium calcium-activated channel subfamily M alpha 1
LAP3	leucine aminopeptidase 3
LDH	lactate dehydrogenase
LDLR	low-density lipoprotein receptor
LEP	leptin
LEPR	leptin receptor
LIPE	lipase E
LKB1	serine-threonine liver kinase B1
LPC	lysophosphatidylcholine
LPE	lysophosphatidylethanolamine
LPL	lipoprotein lipase
LSD-1	lysine-specific demethylase-1
LXR	liver X receptor
MAPKs	mitogen-activated protein kinases
MAT1A2	methionine adenosyltransferase 1A2
MCP1	monocyte chemoattractant protein-1
MDA	malondialdehyde
MDCs	metabolism-disrupting chemicals
MEHP	mono-2-ethylhexyl phthalate
MHINP	monohydroxy isononyl phthalate
MINCH	1,2-cyclohexanedicarboxylic acid mono 4-methyloctyl ester
MMP	mitochondrial membrane potential
MSCs	mesenchymal stem cells
mTOR	mammalian target of rapamycin
MTTP	microsomal triglyceride transfer protein
N6AMT2	N-6 adenine-specific DNA methyltransferase 2
NAFLD	non-alcoholic fatty liver disease
NAMPT	nicotinamide phosphoribosyltransferase
NANOG	nanog homeobox
ND4L	NADH-ubiquinone oxidoreductase subunit 4 L
NDRG1	N-myc downstream regulated 1
NDUFS4	NADH: ubiquinone oxidoreductase subunit S4
NDUS1	NADH dehydrogenase [ubiquinone] iron-sulfur protein 1
NDUS3	NADH dehydrogenase [ubiquinone] iron-sulfur protein 3
NF-κB	nuclear factor kappa-light-chain-enhancer of activated B cells
NO	nitric oxide
NPC2	Niemann-Pick 2
NQO1	NAD(P)H quinone dehydrogenase 1
NRF1	nuclear respiratory factor 1
NRF2	nuclear respiratory factor 2
NRs	nuclear receptors
OA	oleic acid
OCT4	octamer-binding transcription factor 4
OEA	oleoylethanolamide
OGDH	oxoglutarate dehydrogenase
OH-MPHP	6-hydroxy monopropylheptyl phthalate
OPFRs	chlorinated-organophosphorus flame retardants
*p,p’*-DDE	1,1-dichloro-2,2-bis(4-chlorophenyl)ethane
*p,p’*-DDT	1,1,1-trichloro-2,2-bis (p-chlorophenyl)-ethane
pACC	phosphorylated acetyl-CoA carboxylase
p-AKT	phosphorylated v-akt murine thymoma viral oncogene homolog
p-AMPK	phosphorylated AMP-activated protein kinase
PARP	poly (ADP-ribose) polymerase
PBDC1	polysaccharide biosynthesis domain containing 1
PBT	pentabromotoluene
PC	prohormone convertase
PCB-153	polychlorinated biphenyls-153
PCK1	phosphoenolpyruvate carboxykinase 1
PCNA	proliferating cell nuclear antigen
PCs	phosphatidylcholines
PCSK1	proprotein convertase subtilisin/kexin type 1
PDK4	pyruvate dehydrogenase kinase 4
PDX1	pancreatic and duodenal homeobox 1
PE	phosphatidylethanolamine
PEA	palmitoylethanolamide
p-ERK1/2	phosphorylated extracellular signal-regulated protein kinase ½
PFOA	perfluorooctanoic acid
PFOS	perfluorooctanesulfonic acid
PG	phosphatidylglycerol
PHHs	primary human hepatocytes
PI	polyphosphoinositide
p-JNK	phosphorylated c-Jun N-terminal kinase
PKA	protein kinase A
PKB	protein kinase B (also known as AKT)
PKCε	protein kinase C epsilon type
PLIN1	perilipin-1
PLIN2	perilipin 2
PLIN4	perilipin-4
PNPLA2	patatin like phospholipase domain containing 2
PNPLA3	patatin like phospholipase domain containing 3
PPAP2A	phosphatidic acid phosphatase type 2A
PPARGC1A	(or PGC1α) PPARG coactivator 1 alpha
PPARα	peroxisome proliferator-activated receptor α
PPARβ	peroxisome proliferator-activated receptor β
PPARγ	peroxisome proliferator-activated receptor γ
PPARγ1	peroxisome proliferator-activated receptor γ1
PPARγ2	peroxisome proliferator-activated receptor γ2
PPARγC1B	peroxisome proliferator-activated receptor gamma, coactivator 1 beta
PPARδ	peroxisome proliferator-activated receptor δ
PPIA	peptidylprolyl isomerase A
p-PKA	phosphorylated protein kinase A
p-PKC	phosphorylated protein kinase C
PRDM16	PR domain containing 16
PREF-1	adipocyte differentiation-associated protein
PTGS2	prostaglandin-endoperoxide synthase 2
PXR	pregnane X receptor
QpE	quizalofop-p-ethyl
ROS	reactive oxygen species
RUNX2	runt-related transcription factor 2
RXR	retinoid X receptor
RXRα	retinoid X receptor alpha
S100B	calcium binding protein B
SAT	subcutaneous adipose tissue
SCD	stearoyl-CoA desaturase
SCD1	stearoyl-CoA desaturase 1
SCD1A	stearoyl-CoA desaturase 1A
SCD1B	stearoyl-CoA desaturase 1B
SCN9A	sodium voltage-gated channel alpha subunit 9
SCOT	succinyl-CoA-3-oxoacid CoA-transferase
SDHD	succinate dehydrogenase complex subunit D
SGBS	Simpson-Golabi-Behemel syndrome
SIRT1	sirtuin 1
SIRT2	sirtuin 2
SIRT3	sirtuin 3
SIRT5	sirtuin 5
SIRT6	sirtuin 6
SIRT7	sirtuin 7
SM	sphingomyelin
SNAP25	synaptosome-associated protein 25
SOD	superoxide dismutase
SOD1	superoxide dismutase 1
SOD2	superoxide dismutase 2
SOX2	SRY-box 2
SR-A1	scavenger receptor A1
SR-B1	scavenger receptor B1
SRC	spare respiratory capacity
SREBF1	sterol regulatory element binding transcription factor 1
SREBF2	sterol regulatory element binding transcription factor 2
SREBP1	sterol regulatory element-binding protein 1
SREBP1C	sterol regulatory element-binding protein 1c
SREBP2	sterol regulatory element-binding protein 2
STAT3	signal transducer and activator of transcription 3
STAT5	signal transducer and activator of transcription
STAT5A	signal transducer and activator of transcription 5A
STAT5B	signal transducer and activator of transcription 5B
STS	steroid sulfatase
SULT1A1	sulfotransferase family 1A member 1
SULT1A3/4	sulfotransferase family 1A member 3
SUR	sulfonylurea receptor
SUR1	sulfonylurea receptor 1
SVF	stromal vascular fraction
T2DM	type 2 diabetes mellitus
TBB	tetrabromobenzoate
TBBPA	tetrabromobisphenol
TBT	tributyltin
TC	total cholesterol
TCBPA	tetrachlorobisphenol A
TCDD	2,3,7,8-tetrachlorodibenzo-p-dioxin
TCEP	tris (2-chloroethyl) phosphate
TCP	tricresyl phosphate
TCPA	T-complex protein 1 subunit alpha
TCPP	tris (2-chloroisopropyl) phosphate
TCS	triclosan
TDCPP	tris-(2-chloro-1- (chloromethyl) ethyl) phosphate
TERT	telomerase reverse transcriptase
TF	tolylfluanid
TFAM	transcription factor A, mitochondrial
TG	triglyceride
THRSP	thyroid hormone responsive
TIP47	perilipin 3
TMBPF	tetramethyl bisphenol F
TNFα	tumor necrosis factor-α
ToxPi	toxicological Priority Index
TPhP	triphenyl phosphate
TPP	triphenylphosphate
TPT	terpyridine platinum(II) chloride
TR/RXR	thyroid-receptor/retinoid X receptor
TRIB3	tribbles pseudokinase 3
TSPO	translocator protein
UCP-1	uncoupling protein-1
UCP-2	uncoupling protein-2
UCP-3	uncoupling protein-3
UGT2B15	UDP glucuronosyltransferase family 2 member B15
UGTs	UDP-glucuronosyltransferases
UQCRB	ubiquinol-cytochrome C reductase binding protein
VAPA	VAMP associated protein A
VAT	visceral adipose tissue
VDBP	vitamin D-binding protein
VTG1	Vitellogenin 1
WAT	white adipose tissue
WHO	World Health Organization
WT	wild-type
XMEs	xenobiotic-metabolizing enzymes
ZFAND2A	zinc finger AN1-type containing 2A
α2A-AR	adrenergic receptor α2A
β-AR	β adrenergic receptor
β-TC-6	beta-tumor cell-6
